# Machine learning algorithms for individualized prediction of prognosis in breast cancer liver metastases and the prognostic impact of primary tumor surgery: a multicenter study

**DOI:** 10.3389/fendo.2025.1656191

**Published:** 2025-10-13

**Authors:** Chunmei Chen, Jundong Wu, Bo Xu, Weiwen Li, Chengming Zhong, Zhibing Yan, Qipeng Zhong, Ronggang Li, Mingtao Shao, Yan Dong, Yutong Fang, Yong Li, Qunchen Zhang

**Affiliations:** ^1^ Department of Breast, Jiangmen Central Hospital, Jiangmen, Guangdong, China; ^2^ The Breast Center, Cancer Hospital of Shantou University Medical College, Shantou, Guangdong, China; ^3^ Department of General Surgery, The First Affiliated Hospital of Jinan University, Guangzhou, Guangdong, China; ^4^ Department of General Surgery, Guangzhou First People’s Hospital, School of Medicine, South China University of Technology, Guangzhou, Guangdong, China; ^5^ Department of Equipment, Jiangmen Xinhui Maternal and Child Health Hospital, Jiangmen, Guangdong, China; ^6^ Department of Anesthesiology, Jiangmen Central Hospital, Jiangmen, Guangdong, China; ^7^ Department of Pathology, Jiangmen Central Hospital, Jiangmen, Guangdong, China

**Keywords:** breast cancer liver metastases, machine learning, prognosis, surgery, random forest

## Abstract

**Background:**

The prognosis of patients with breast cancer liver metastasis (BCLM) is generally poor, and there are no specific treatment guidelines. Accurate prognostic tools are needed to estimate survival and support individualized management.

**Methods:**

The study cohort consisted of 4,817 patients diagnosed with BCLM from the SEER database spanning 2010 to 2020. Candidate predictors were screened using univariate and multivariate Cox regression. Five machine-learning algorithms—Random Forest (RF), Logistic Regression, XGBoost, Decision Tree, and Gradient Boosting—were trained to predict 6-month, 1-, 3-, and 5-year overall survival (OS) and breast cancer–specific survival (BCSS). Labels at each horizon were handled with inverse probability-of-censoring weighting, and performance was assessed with IPCW-AUC, accuracy, F1 score, calibration, and decision curve analysis. External validation included 124 BCLM patients from two Chinese hospitals. To evaluate the effect of primary tumor surgery (PTS), we modeled PTS as a time-dependent exposure and performed time-dependent Cox analyses with time-varying effects, Simon–Makuch curves, piecewise Cox modeling, 2-month landmark analysis, and E-value calculations.

**Results:**

RF was the top performer for both OS and BCSS across horizons (training AUCs = 0.840–0.899; internal test AUCs = 0.763–0.787), with good calibration and net benefit. External validation showed consistent discrimination (AUCs 0.779–0.815). SHAP analyses highlighted chemotherapy, age, subtype, and surgery as dominant contributors. In time-dependent analyses, PTS was associated with reduced risks of death (OS: HR 0.80, 95% CI 0.72–0.88) and breast cancer–specific mortality (BCSS: HR 0.77, 95% CI 0.69–0.86); findings were directionally consistent in piecewise and landmark analyses, and E-values (≥1.81) supported robustness to moderate unmeasured confounding.

**Conclusion:**

We developed and externally validated robust RF-based models for predicting OS and BCSS in BCLM. Our results indicate that PTS is associated with longer survival and lower breast cancer-specific mortality in carefully selected patients, supporting consideration within individualized, multidisciplinary decision-making.

## Introduction

Breast cancer (BC) is increasingly becoming the most common cancer worldwide and is the primary cause of cancer-related mortality among women (1). Common sites of metastasis in advanced BC include the bones, lungs, liver, and brain (2), with liver metastases occurring in 50–61% of cases (3). Although the liver is not the organ with the highest probability of metastasis, involvement of the liver is an independent predictor of worse overall survival (OS) in BC patients, compared to factors such as bone metastasis (2). Once liver metastasis occurs, the prognosis is generally poor, with a median survival of only 4–8 months without treatment. For patients who respond to systemic treatment, the median survival from the date of diagnosis is only 18–24 months, with most patients showing disease progression after approximately 1–2 years of stable treatment (4). The 5-year and 10-year survival rates are as low as 27% and 13%, respectively (5, 6). Currently, there are no specific treatment guidelines for patients with breast cancer liver metastases (BCLM) (7). Furthermore, various clinical characteristics significantly influence the prognosis of BCLM patients (8). Therefore, there is an urgent need for precise prognostic models to assess the survival of BCLM patients and to assist in optimizing their individualized management.

Machine learning (ML) algorithms have emerged as pivotal tools in the field of medicine, notably in the construction of models that predict patient outcomes with high accuracy (9–11). Ranging from conventional regression models to advanced deep learning architectures, ML excels in processing extensive, complex datasets by identifying non-linear relationships between inputs and outcomes (12). The capacity of ML to consolidate and analyze various forms of clinical data enables the provision of more accurate and personalized care for patients (13, 14). Despite its extensive application in cancer prognostics, the potential of ML to forecast outcomes for BCLM patients remains largely unexplored. The individualized identification of these high-risk BCLM patients could significantly influence our clinical decisions and lead to the creation of tailored therapeutic strategies.

The role of primary tumor surgery (PTS) and its approaches remains controversial in the management of patients initially diagnosed with stage IV BC. Several retrospective studies have highlighted that appropriately selected subsets of patients—such as those with smaller metastatic burdens, positive hormone receptors, and younger ages—may benefit in terms of OS from PTS (15–20). The MF07–01 trial further demonstrated significant survival benefits for patients who underwent PTS followed by comprehensive systemic treatments (21). However, other prospective randomized controlled trials have shown that primary tumor surgery does not confer an OS advantage in stage IV patients, except in carefully selected subgroups (22–24). Typically, the local treatment of BCLM patients focuses more on the management of liver metastases, and the benefits of PTS based on comprehensive systemic therapy remains uncertain and under debate.

In this study, we utilized the Surveillance, Epidemiology, and End Results (SEER) database to develop models predicting OS and breast cancer-specific survival (BCSS) in BCLM patients, using five different ML algorithms. We also retrospectively collected data from two Chinese hospitals on BCLM patients to evaluate the applicability of these models. To further investigate the prognostic impact of PTS in BCLM patients, we applied time-dependent Cox regression models and conducted multiple sensitivity analyses. The aim of this study is to provide robust, individualized survival predictions and to explore the potential survival benefits of PTS to inform clinical decision-making in BCLM management.

## Materials and methods

### Patients and study design

**Figure 1:**
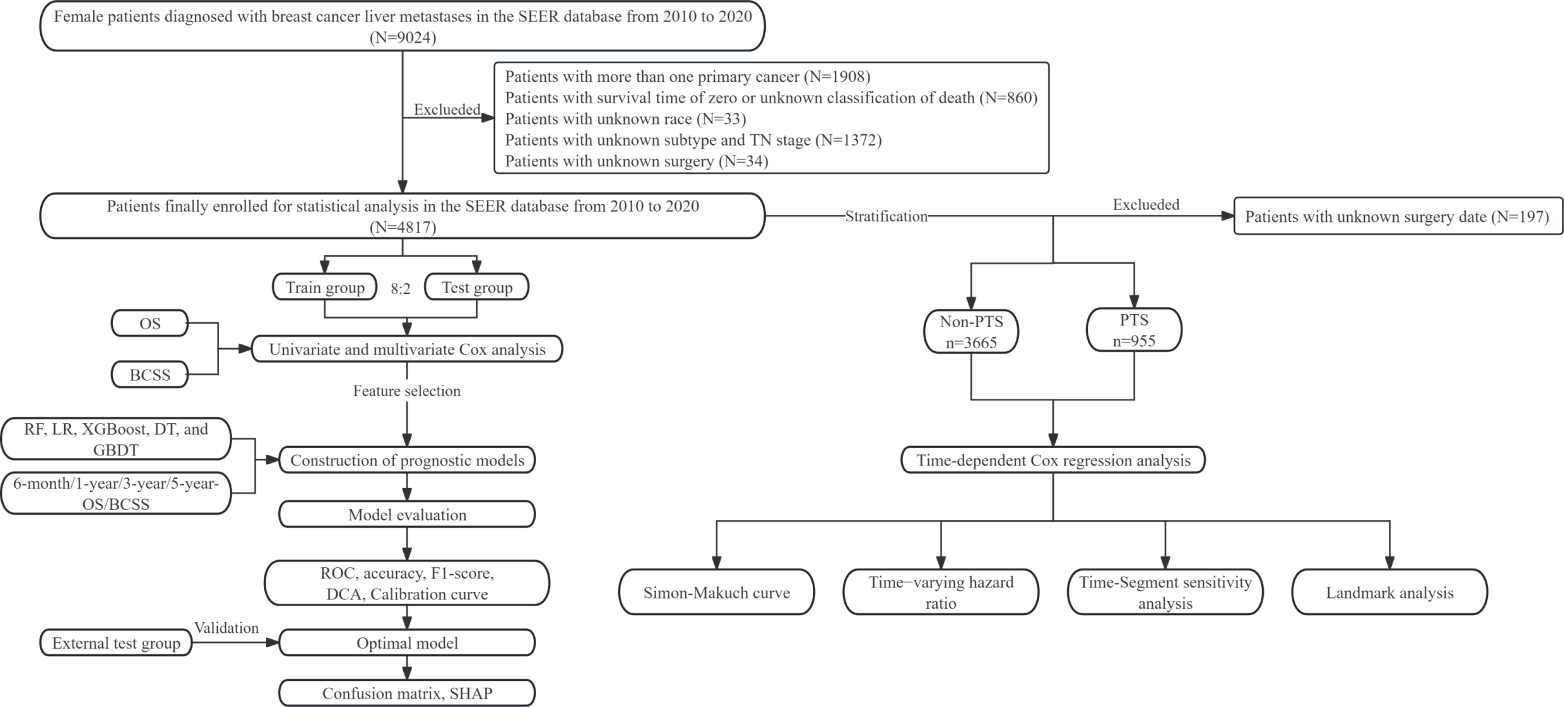
Flow chart of this study. SEER, surveillance, epidemiology, and end results; OS, overall survival; BCSS, breast cancer-specific survival; RF, random forest; LR, logistic regression; XGBoost, extreme gradient boosting; DT, decision tree; GBDT, gradient boosting decision tree; ROC, receiver operating characteristic; DCA, decision curve analysis; SHAP, SHapley Additive exPlanations; PTS, primary tumor surgery.

This study adhered to the Transparent Reporting of a multivariable prediction model for Individual Prognosis Or Diagnosis (TRIPOD + AI) checklist (25). The flowchart illustrates our study design (**Figure 1**). This study encompassed three patient cohorts. The SEER database, a publicly accessible repository, is established and maintained by the National Cancer Institute. Initially, data from the SEER 17 registries research data [(2000–2020); version 8.4.3] were utilized. Given that Human epidermal growth factor receptor 2 (HER2) status and specific metastatic organ sites were incorporated from 2010 onwards, the inclusion criteria were as follows: (1) female sex; (2) diagnosis year falling within 2010 to 2020; (3) anatomical site and morphological coding aligned with the International Classification of Diseases Oncology Third Edition (ICD-O-3); (4) concurrent liver metastases at breast cancer diagnosis; and (5) survival time >0 months. Patients with multiple primary tumors were excluded. Furthermore, retrospective data were gathered from BCLM patients treated at Jiangmen Central Hospital (JCH) and Cancer Hospital of Shantou University Medical College (CHSU) between January 2010 and June 2023. This study was approved by the Ethics Committees of JCH (No. 2024312) and CHSU (No. 2024216). Our Ethics Committees, in accordance with the Guidelines for Ethical Review Committees of Clinical Research Involving Humans (2023 edition), determined that informed consent was not required and thus approved the waiver of informed consent.

### Data collection

The following patient characteristics were obtained: age, race, marital status, median household income (inflation adjusted), histological type, tumor location, histologic grade, molecular subtype, T stage, N stage, surgery, radiotherapy, chemotherapy, bone metastases, brain metastases, and lung metastases. Tumor-related variables comprised histological type, coded according to the ICD-O-3; primary tumor location, based on ICD-O-3 topography codes; and histological grade. Subtype was determined using available molecular or clinicopathological surrogates (hormone receptor and HER2 status). Tumor stage was assessed using the American Joint Committee on Cancer (AJCC) TNM staging system (8th edition). The primary endpoint was OS and the secondary endpoint was BCSS. The median follow-up time was 56 months (IQR: 28.0–86.0) for patients from the SEER database and 35 months (20.0–58.0) for patients from two hospitals in China. Survival rates and event counts for each prediction interval are presented in **Supplementary Table S1**.

### Feature selection, model construction and evaluation

In order to reduce redundant variables and model overfitting, we employed univariate and multivariate Cox regression analyses within each training fold to select independent factors associated with prognosis in the training group. Statistically significant features were utilized for subsequent ML model construction. Five commonly used ML algorithms, including Random Forest (RF), Logistic Regression (LR), XGBoost, Decision Tree (DT), and Gradient Boosting Decision Tree (GBDT), were applied to predict 6-month, 1-year, 3-year, and 5-year Overall Survival (OS) and Breast Cancer-Specific Survival (BCSS). For each prediction horizon t ∈{0.5,1,3,5} year, we defined a binary outcome ${\text{Y}}_{t}$ = 1 if the event occurred by t  and ${\text{Y}}_{t}$ = 0 otherwise. Patients censored prior to t had unknown labels and were handled using inverse probability-of-censoring weighting (IPCW). The censoring survival function $\hat{G}(t)$ was estimated via the Kaplan–Meier method, with right-censoring defined as alive at last follow-up. Each labeled observation received weight:


$$w_{i}(t)=\frac{1}{\hat{G}(\min{\left(T_{i},t\right)}^{-})}$$

, i.e., at the time the label became known. Classifiers were then trained with these weights and evaluated using IPCW-weighted Area Under the Curve (AUC) and Brier score at each prediction horizon (26). RF is a non-parametric machine learning algorithm based on ensemble learning, which predicts by constructing multiple decision trees and integrating them through voting or averaging. Each decision tree is generated by bootstrapping the training set, with only a random subset of features considered for splitting at each node. This randomness effectively reduces model variance and enhances generalization performance. LR is a generalized linear model that estimates probabilities by mapping the output of a linear function to a sigmoid function and estimates model parameters by maximizing the likelihood function. XGBoost is an efficient gradient boosting decision tree algorithm that iteratively trains decision tree models by optimizing the negative gradient of the loss function and utilizes regularization techniques to control model complexity. DT is a classification and regression method based on tree structure, which partitions the dataset into different categories or values through a series of decision nodes. GBDT is an algorithm that enhances model performance by iteratively training decision trees and optimizing the loss function. It constructs decision trees sequentially and trains each subsequent tree using the residual of the previous tree, effectively reducing model bias.

To enhance model robustness, hyperparameters were optimized via grid search with 10-fold cross-validation. Patients from the SEER database were randomly divided into training and internal testing groups at an 8:2 ratio. Patients from two Chinese hospitals were used as an external testing group to further validate the generalizability of the optimal model. The performance of ML models was evaluated using AUC, accuracy, and F1 score of the training and testing groups. Calibration curves were used to assess the accuracy and reliability of model predictions. Decision Curve Analysis (DCA) was employed to determine the clinical utility of the models. Confusion matrices intuitively displayed the classification performance of the models. SHAPley Additive exPlanations (SHAP) values were computed using the “shap” library to explain the contribution or importance of each feature to the model.

### PTS

To minimize immortal-time bias and avoid exposure misclassification around surgery, PTS was modeled as a time-dependent covariate. For patients undergoing surgery, follow-up time before surgery was classified as “non-PTS,” and time after surgery as “PTS.” Patients with a surgical history but no recorded surgery date were excluded from the main analysis, as their exposure periods could not be reliably defined. We performed multivariable analyses using a time-dependent Cox regression model based on PTS, incorporating an interaction term with log(time) to examine time-varying effects, and generated hazard ratio (HR) curves over time. For descriptive visualization, Simon–Makuch curves were additionally plotted. A segmented Cox model was applied with the follow-up time divided at 24 months, and a 2-month landmark analysis was conducted among patients who survived beyond this time point. To evaluate robustness against unmeasured confounding, the E-value was calculated using the following formula:


$$\text{E}={\text{HR}}^{*}+\sqrt{{\text{HR}}^{*}{\times \text{(HR}}^{*}-1)}\text{,if HR}<1,\;{\text{HR}}^{*}=\frac{1}{\text{HR}}$$

### Statistical analysis

Categorical data were presented as counts and percentages and were compared using either the Chi-squared test or Fisher’s exact test depending on the sample size. Kaplan-Meier survival analysis was conducted using the log-rank test. Univariate and multivariate Cox regression analyses were employed to identify modeling features. The generalized variance inflation factor (GVIF) was utilized to detect multicollinearity in the Cox regression model. An adjusted GVIF value less than 2 was considered acceptable. Statistical analyses were performed using R software version 4.2.1 (r-project.org/) or Python version 3.8 (Python Software Foundation). A significance level of P< 0.05 indicated statistical significance.

## Results

### Clinicopathologic characteristics

Finally, we collected data on 4817 BCLM patients who met the eligibility criteria from the SEER database. As shown in **Table 1**, 1545 patients (32.07%) were aged 50 years or younger, 1945 patients (40.38%) were between the ages of 51 and 65, and 1327 patients (27.55%) were 66 years or older. The majority of patients were Caucasian (72.33%). Approximately 45.84% of the patients were married, and 24.60% were single or homosexual. In terms of household income, 54.35% of the patients had incomes exceeding $70,000. About 80% of the patients were diagnosed with invasive ductal carcinoma (IDC), and 25.76% had tumors located in the upper outer quadrant. Patients classified as G2 and G3 accounted for 22.69% and 36.64%, respectively, while only 2.47% were G1. The molecular subtype HR+/HER2- was present in 44.2% of patients, with dual-positive and dual-negative subtypes accounting for 24.02% and 15.11%, respectively. The distribution of T stages from T1 to T4 was 11.21%, 34.57%, 18.21%, and 36.02%, and for N stages from N0 to N3 was 19.27%, 53.35%, 12.21%, and 15.18%, respectively. Bone metastases or lung metastases were present in 59.54% and 32.74% of patients, respectively, with only 8.95% having brain metastases. Regarding treatment, 23.92% of patients underwent primary tumor surgery, 27.40% received radiotherapy, and the majority (75.77%) received chemotherapy. Additionally, 124 BCLM patients from the JCH and CHSU cohorts were included as the external group. In contrast to the SEER cohort, all patients in the external cohorts were Asian, and only 8.06% underwent surgery.

**Table 1:** Baseline characteristics of patients with breast cancer liver metastases.

Variables	SEER cohort	External cohort
Age		
≤50	1545 (32.07)	38 (30.65)
51-65	1945 (40.38)	56 (45.16)
≥66	1327 (27.55)	30 (24.19)
Race		
White	3484 (72.33)	0 (0.00)
Black	843 (17.50)	0 (0.00)
Others	490 (10.17)	124 (100.00)
Marital status		
Married	2208 (45.84)	73 (58.87)
Singled/homosexual	1185 (24.60)	24 (19.35)
Widow/divorced/others	1424 (29.56)	27 (21.77)
Median household income (inflation adjusted)		
<$40,000	161 (3.34)	15 (12.10)
$40,000-59,999	1034 (21.47)	26 (20.97)
$60,000-69,999	1004 (20.84)	52 (41.94)
$70,000+	2618 (54.35)	31 (25.00)
Histological type		
Invasive ductal carcinoma	3841 (79.74)	103 (83.06)
Invasive lobular carcinoma	280 (5.81)	7 (5.65)
Mixed	235 (4.88)	3 (2.42)
Other	461 (9.57)	11 (8.87)
Tumor location		
Upper outer	1241 (25.76)	31 (25.00)
Lower outer	258 (5.36)	5 (4.03)
Lower inner	177 (3.67)	4 (3.23)
Upper inner	311 (6.46)	4 (3.23)
Central	286 (5.94)	7 (5.65)
Others	2544 (52.81)	73 (58.87)
Grade		
G1	119 (2.47)	1 (0.81)
G2	1093 (22.69)	27 (21.77)
G3	1765 (36.64)	31 (25.00)
Unknown	1840 (38.20)	65 (52.42)
Subtype		
HR+/HER2-	2129 (44.20)	58 (46.77)
HR+/HER2+	1157 (24.02)	35 (28.23)
HR-/HER2+	803 (16.67)	21 (16.94)
HR-/HER2-	728 (15.11)	10 (8.06)
T stage		
T1	540 (11.21)	5 (4.03)
T2	1665 (34.57)	48 (38.71)
T3	877 (18.21)	27 (21.77)
T4	1735 (36.02)	44 (35.48)
N stage		
N0	928 (19.27)	17 (13.71)
N1	2570 (53.35)	43 (34.68)
N2	588 (12.21)	30 (24.19)
N3	731 (15.18)	34 (27.42)
Surgery		
No	3665 (76.08)	114 (91.94)
Yes	1152 (23.92)	10 (8.06)
Radiotherapy		
No/unknown	3497 (72.60)	98 (79.03)
Yes	1320 (27.40)	26 (20.97)
Chemotherapy		
No/unknown	1167 (24.23)	30 (24.19)
Yes	3650 (75.77)	94 (75.81)
Bone metastases		
No/unknown	1949 (40.46)	44 (35.48)
Yes	2868 (59.54)	80 (64.52)
Brain metastases		
No/unknown	4386 (91.05)	109 (87.90)
Yes	431 (8.95)	15 (12.10)
Lung metastases		
No/unknown	3240 (67.26)	90 (72.58)
Yes	1577 (32.74)	34 (27.42)

SEER, Surveillance, Epidemiology, and End Results.

### Feature selection

We analyzed the correlation between variables and generated a heatmap, which indicated no multicollinearity among the variables (**Supplementary Figure S1**). For clarity, the Cox regression results presented in **Table 2** were performed on the overall training cohort to illustrate variable associations, while per-fold selections were used internally during cross-validation. Univariate Cox regression analysis revealed that age, race, marital status, median household income, histological type, tumor location, subtype, T stage, N stage, surgery, chemotherapy, bone metastases, brain metastases, and lung metastases significantly affected OS. Meanwhile, significantly affecting BCSS were age, race, marital status, median household income, histological type, subtype, tumor location, T stage, N stage, surgery, chemotherapy, bone metastases, brain metastases, and lung metastases.

**Table 2:** Univariate and multivariate Cox analyses of patients with breast cancer liver metastases in the SEER database.

	Univariate Cox analysis						Multivariate Cox analysis					
	OS			BCSS			OS			BCSS		
	HR	95%CI	P	HR	95%CI	P	HR	95%CI	P	HR	95%CI	P
Age												
≤50		Reference			Reference			Reference			Reference	
51-65	1.43	1.32-1.56	<.001	1.44	1.32-1.58	<.001	1.24	1.14-1.35	<.001	1.25	1.14-1.36	<.001
≥66	2.11	1.93-2.32	<.001	2.04	1.85-2.24	<.001	1.59	1.44-1.76	<.001	1.53	1.38-1.70	<.001
Race												
White		Reference			Reference			Reference			Reference	
Black	1.31	1.20-1.44	<.001	1.30	1.18-1.42	<.001	1.28	1.16-1.40	<.001	1.26	1.15-1.39	<.001
Others	0.84	0.74-0.95	0.007	0.83	0.73-0.95	0.006	0.94	0.83-1.07	0.334	0.93	0.81-1.06	0.279
Marital status												
Married		Reference			Reference			Reference			Reference	
Singled/homosexual	1.14	1.04-1.24	0.004	1.13	1.03-1.24	0.009	1.05	0.96-1.15	0.278	1.04	0.95-1.14	0.397
Widow/divorced/others	1.42	1.31-1.54	<.001	1.41	1.30-1.53	<.001	1.12	1.03-1.22	0.010	1.12	1.02-1.22	0.014
Median household income inflation adjusted												
<$40,000		Reference			Reference			Reference			Reference	
$40,000-59,999	0.88	0.73-1.07	0.199	0.92	0.76-1.12	0.421	1.02	0.84-1.23	0.873	1.06	0.87-1.29	0.569
$60,000-69,999	0.74	0.61-0.89	0.002	0.78	0.64-0.95	0.014	0.89	0.73-1.08	0.225	0.94	0.77-1.15	0.544
$70,000+	0.62	0.52-0.74	<.001	0.65	0.54-0.79	<.001	0.76	0.63-0.91	0.003	0.80	0.66-0.97	0.023
Histological type												
Invasive ductal carcinoma		Reference			Reference			Reference			Reference	
Invasive lobular carcinoma	1.12	0.97-1.30	0.130	1.10	0.94-1.28	0.229	1.00	0.86-1.17	0.978	0.98	0.84-1.15	0.847
Mixed	0.95	0.81-1.12	0.555	0.94	0.79-1.11	0.446	1.01	0.86-1.19	0.902	0.99	0.84-1.17	0.905
Other	1.36	1.21-1.52	<.001	1.38	1.23-1.54	<.001	1.15	1.03-1.29	0.017	1.16	1.04-1.31	0.011
Tumor location												
Upper outer		Reference			Reference			Reference			Reference	
Lower outer	1.04	0.88-1.23	0.676	1.03	0.86-1.22	0.748	1.16	0.98-1.38	0.078	1.16	0.97-1.38	0.103
Lower inner	0.90	0.74-1.10	0.304	0.91	0.74-1.12	0.386	0.95	0.78-1.16	0.613	0.96	0.78-1.19	0.726
Upper inner	0.98	0.84-1.15	0.836	0.97	0.82-1.14	0.721	0.97	0.83-1.13	0.688	0.95	0.81-1.12	0.573
Central	1.13	0.96-1.32	0.137	1.15	0.98-1.35	0.091	0.99	0.84-1.16	0.868	1.01	0.86-1.19	0.920
Others	1.16	1.07-1.26	<.001	1.16	1.06-1.26	<.001	1.09	1.01-1.19	0.040	1.09	1.00-1.19	0.062
Grade												
G1		Reference			Reference			Reference			Reference	
G2	0.94	0.75-1.16	0.538	0.96	0.76-1.20	0.692	/	/	/	/	/	/
G3	1.17	0.95-1.44	0.146	1.21	0.97-1.51	0.084	/	/	/	/	/	/
Unknown	1.19	0.97-1.48	0.101	1.22	0.97-1.52	0.085	/	/	/	/	/	/
Subtype												
HR+/HER2-		Reference			Reference			Reference			Reference	
HR+/HER2+	0.58	0.53-0.63	<.001	0.58	0.53-0.64	<.001	0.71	0.64-0.78	<.001	0.71	0.64-0.78	<.001
HR-/HER2+	0.68	0.62-0.76	<.001	0.69	0.62-0.77	<.001	0.91	0.81-1.01	0.080	0.91	0.81-1.02	0.104
HR-/HER2-	2.05	1.86-2.26	<.001	2.08	1.89-2.29	<.001	2.57	2.32-2.85	<.001	2.62	2.36-2.91	<.001
T stage												
T1		Reference			Reference			Reference			Reference	
T2	1.06	0.94-1.20	0.352	1.06	0.93-1.20	0.384	1.11	0.98-1.26	0.097	1.11	0.97-1.26	0.115
T3	1.15	1.01-1.32	0.043	1.16	1.01-1.34	0.036	1.12	0.97-1.29	0.110	1.13	0.98-1.30	0.097
T4	1.48	1.31-1.67	<.001	1.49	1.31-1.68	<.001	1.29	1.14-1.47	<.001	1.30	1.14-1.48	<.001
N stage												
N0		Reference			Reference			Reference			Reference	
N1	0.84	0.77-0.92	<.001	0.84	0.76-0.92	<.001	0.85	0.77-0.93	<.001	0.84	0.76-0.92	<.001
N2	0.87	0.77-0.98	0.028	0.85	0.75-0.96	0.012	0.90	0.80-1.03	0.129	0.88	0.77-1.00	0.059
N3	0.93	0.83-1.05	0.253	0.94	0.83-1.06	0.305	0.89	0.78-1.00	0.051	0.88	0.78-1.00	0.053
Surgery												
No		Reference			Reference			Reference			Reference	
Yes	0.60	0.55-0.65	<.001	0.59	0.54-0.64	<.001	0.70	0.64-0.76	<.001	0.69	0.63-0.76	<.001
Radiotherapy												
No/unknown		Reference			Reference			Reference			Reference	
Yes	0.97	0.90-1.05	0.458	0.99	0.91-1.07	0.741	/	/	/	/	/	/
Chemotherapy												
No/unknown		Reference			Reference			Reference			Reference	
Yes	0.47	0.44-0.51	<.001	0.48	0.44-0.52	<.001	0.55	0.50-0.59	<.001	0.55	0.50-0.59	<.001
Bone metastases												
No/unknown		Reference			Reference			Reference			Reference	
Yes	1.44	1.34-1.55	<.001	1.48	1.37-1.59	<.001	1.29	1.20-1.40	<.001	1.32	1.22-1.43	<.001
Brain metastases												
No/unknown		Reference			Reference			Reference			Reference	
Yes	1.82	1.62-2.04	<.001	1.83	1.63-2.06	<.001	1.61	1.43-1.81	<.001	1.60	1.42-1.80	<.001
Lung metastases												
No/unknown		Reference			Reference			Reference			Reference	
Yes	1.62	1.51-1.74	<.001	1.63	1.51-1.75	<.001	1.28	1.19-1.39	<.001	1.29	1.19-1.39	<.001

OS, overall survival; BCSS, breast cancer-specific survival; HR, hazard ratio; CI, confidence interval.

Multivariate Cox regression analysis was employed to further explore independent risk factors affecting prognosis. GVIF suggested that there was no multicollinearity between the variables in the regression model (**Supplementary Table S2**). Older age, other pathological type and tumor location, HR-/HER2-, T4 stage, and the presence of other distant metastases were associated with poorer OS. Socially, Black and divorced patients had worse OS, whereas patients with household income exceeding $70,000 exhibited better OS. Similarly, patients who underwent surgery and radiotherapy demonstrated improved OS. Moreover, factors indicating worse BCSS included older age, being black, being divorced, other pathological types, HR-/HER2-, T4 stage, and the presence of other distant metastases. In contrast, household income over $70,000, HR+/HER2+, N1 stage, and having undergone surgery and chemotherapy were associated with better BCSS.

### Establishment and evaluation of prognostic models

**Figure 2:**
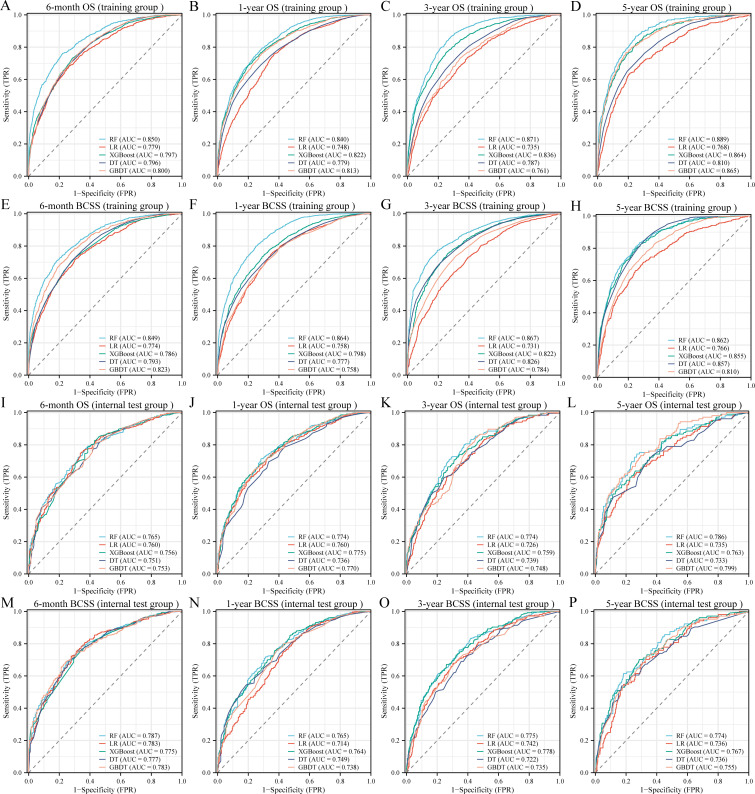
Receiver operating characteristic curves of five machine learning models in the training **(A-H)** and internal test **(I-P)** groups. OS, overall survival; BCSS, breast cancer-specific survival; AUC, area under the curve; RF, random forest; LR, logistic regression; XGBoost, extreme gradient boosting; DT, decision tree; GBDT, gradient boosting decision tree.

We incorporated significant features into subsequent ML model construction to predict the 6-month, 1-year, 3-year, and 5-year OS and BCSS for BCLM patients. The performance of five ML models in the training and internal test groups was depicted in **Figure 2**. The RF models demonstrated excellent performance in predicting 6-month OS (Training: AUC=0.850; Internal Test: AUC=0.765), 1-year OS (Training: AUC=0.840; Internal Test: AUC=0.774), 3-year OS (Training: AUC=0.871; Internal Test: AUC=0.774), and 5-year OS (Training: AUC=0.889; Internal Test: AUC=0.786). Additionally, the RF models also performed well in predicting BCSS at 6 months (Training: AUC=0.849; Internal Test: AUC=0.787), 1 year (Training: AUC=0.864; Internal Test: AUC=0.765), 3 years (Training: AUC=0.867; Internal Test: AUC=0.775), and 5 years (Training: AUC=0.862; Internal Test: AUC=0.774). Other ML models such as LR (6-month OS: AUC=0.779; 1-year OS: AUC = 0.748; 3-year OS: AUC = 0.735; 5-year OS: AUC = 0.768; 6-month BCSS: AUC=0.774; 1-year BCSS: AUC = 0.758; 3-year BCSS: AUC = 0.731; 5-year BCSS: AUC = 0.766), XGBoost (6-month OS: AUC=0.797; 1-year OS: AUC = 0.822; 3-year OS: AUC = 0.836; 5-year OS: AUC = 0.864; 6-month BCSS: AUC=0.786; 1-year BCSS: AUC = 0.798; 3-year BCSS: AUC = 0.822; 5-year BCSS: AUC = 0.855), DT (6-month OS: AUC=0.796; 1-year OS: AUC = 0.779; 3-year OS: AUC = 0.787; 5-year OS: AUC = 0.810; 6-month BCSS: AUC=0.793; 1-year BCSS: AUC = 0.777; 3-year BCSS: AUC = 0.826; 5-year BCSS: AUC = 0.857), and GBDT (6-month OS: AUC=0.800; 1-year OS: AUC = 0.813; 3-year OS: AUC = 0.761; 5-year OS: AUC = 0.865; 6-month BCSS: AUC=0.823; 1-year BCSS: AUC = 0.758; 3-year BCSS: AUC = 0.784; 5-year BCSS: AUC = 0.810) showed slightly inferior performance compared to RF in the training group. Moreover, the RF models’ accuracy (6-month OS: 0.776 and 0.745; 1-year OS: 0.761 and 0.714; 3-year OS: 0.764 and 0.711; 5-year OS: 0.807 and 0.732; 6-month BCSS: 0.793 and 0.759; 1-year BCSS: 0.782 and 0.708; 3-year BCSS: 0.784 and 0.714; 5-year BCSS: 0.759 and 0.786) and F1-scores (6-month OS: 0.615 and 0.498; 1-year OS: 0.686 and 0.650; 3-year OS: 0.828 and 0.768; 5-year OS: 0.863 and 0.823; 6-month BCSS: 0.600 and 0.526; 1-year BCSS: 0.703 and 605; 3-year BCSS:0.832 and 0.736; 5-year BCSS: 0.841 and 0.865) in both the training and internal testing groups were satisfactory (**Table 3**).

**Table 3:** Performance of machine learning prognostic models in the training and internal test groups.

Survival	Indicators	Training group					Internal test group				
		RF	LR	XGBoost	DT	GBDT	RF	LR	XGBoost	DT	GBDT
6-month OS	AUC	0.850	0.779	0.797	0.796	0.800	0.765	0.760	0.756	0.751	0.753
	Accuracy	0.776	0.736	0.718	0.713	0.700	0.745	0.730	0.748	0.755	0.752
	F1‐score	0.615	0.524	0.532	0.536	0.506	0.498	0.505	0.500	0.434	0.543
1-year OS	AUC	0.840	0.748	0.822	0.779	0.813	0.774	0.760	0.775	0.736	0.770
	Accuracy	0.761	0.714	0.727	0.700	0.744	0.714	0.704	0.678	0.685	0.688
	F1‐score	0.686	0.603	0.673	0.636	0.662	0.650	0.628	0.648	0.602	0.634
3-year OS	AUC	0.871	0.735	0.836	0.787	0.761	0.774	0.726	0.759	0.739	0.748
	Accuracy	0.764	0.649	0.755	0.699	0.682	0.711	0.692	0.695	0.703	0.644
	F1‐score	0.828	0.734	0.713	0.770	0.752	0.768	0.770	0.749	0.780	0.660
5-year OS	AUC	0.889	0.768	0.864	0.810	0.865	0.786	0.735	0.763	0.733	0.799
	Accuracy	0.807	0.766	0.761	0.785	0.769	0.732	0.750	0.604	0.644	0.710
	F1‐score	0.863	0.843	0.865	0.821	0.871	0.823	0.839	0.702	0.741	0.800
6-month BCSS	AUC	0.849	0.774	0.786	0.793	0.823	0.787	0.783	0.775	0.777	0.783
	Accuracy	0.793	0.702	0.701	0.716	0.729	0.759	0.722	0.733	0.707	0.700
	F1‐score	0.600	0.511	0.523	0.526	0.563	0.526	0.503	0.481	0.504	0.514
1-year BCSS	AUC	0.864	0.758	0.798	0.777	0.758	0.765	0.714	0.764	0.749	0.738
	Accuracy	0.782	0.720	0.724	0.695	0.723	0.708	0.724	0.715	0.633	0.709
	F1‐score	0.703	0.608	0.637	0.625	0.591	0.605	0.500	0.567	0.611	0.586
3-year BCSS	AUC	0.867	0.731	0.822	0.826	0.784	0.775	0.742	0.778	0.722	0.735
	Accuracy	0.784	0.691	0.748	0.721	0.721	0.714	0.690	0.694	0.660	0.679
	F1‐score	0.832	0.724	0.805	0.788	0.772	0.736	0.731	0.764	0.706	0.719
5-year BCSS	AUC	0.862	0.766	0.855	0.857	0.810	0.774	0.736	0.767	0.736	0.755
	Accuracy	0.759	0.74	0.759	0.72	0.713	0.786	0.679	0.713	0.784	0.673
	F1‐score	0.841	0.818	0.864	0.825	0.814	0.865	0.779	0.807	0.874	0.775

OS, overall survival; BCSS, breast cancer-specific survival; AUC, area under the curve; RF, random forest; LR, logistic regression; XGBoost, extreme gradient boosting; DT, decision tree; GBDT, gradient boosting decision tree.

**Figure 3:**
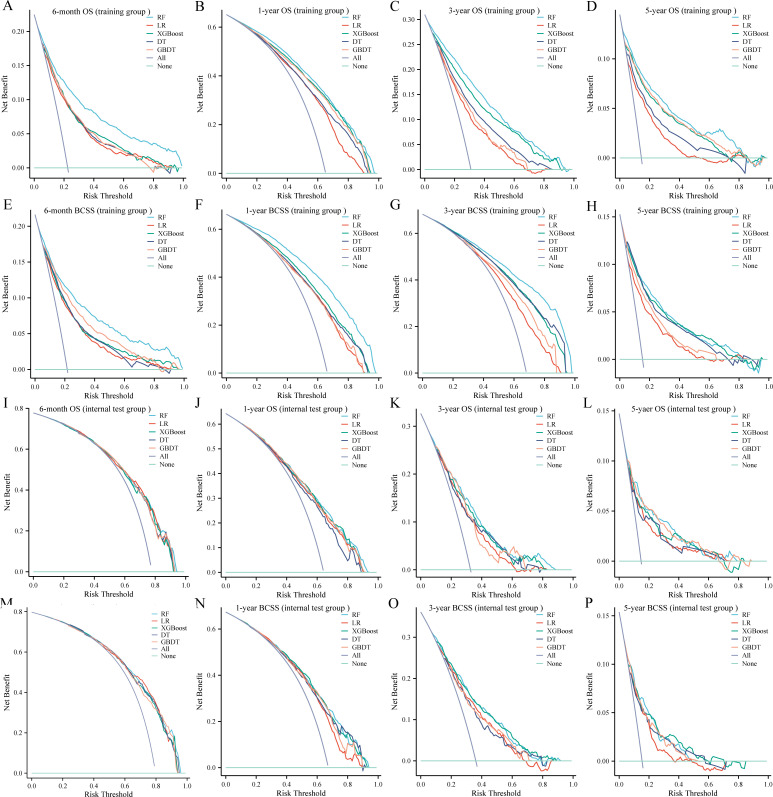
Decision curves for the five machine learning models in the training **(A-H)** and internal test **(I-P)** groups. OS, overall survival; BCSS, breast cancer-specific survival; RF, random forest; LR, logistic regression; XGBoost, extreme gradient boosting; DT, decision tree; GBDT, gradient boosting decision tree.

**Figure 4:**
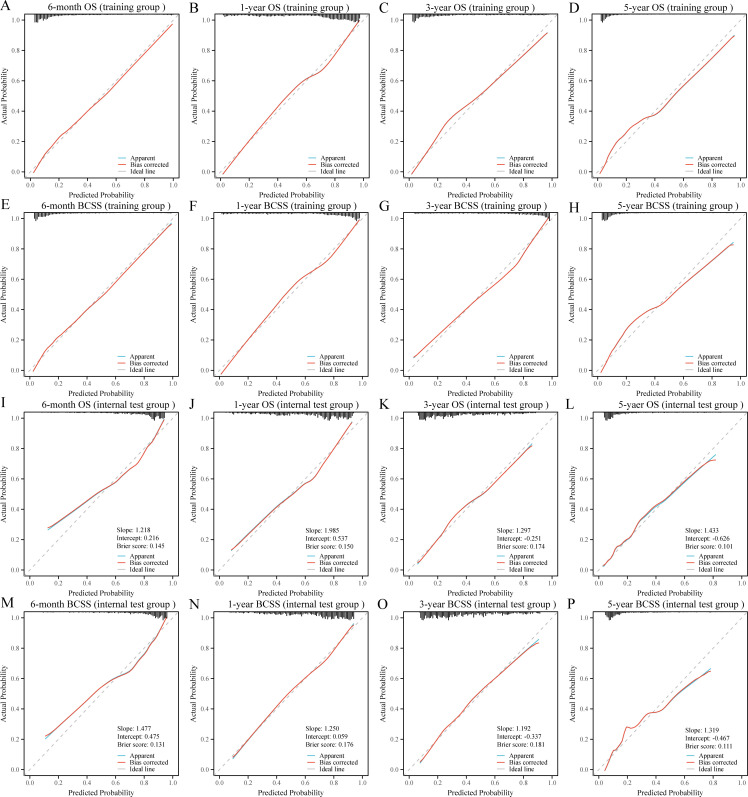
Calibration curves for the random forest models in the training **(A-H)** and internal test **(I-P)** groups. OS, overall survival; BCSS, breast cancer-specific survival.

DCA was used to evaluate the clinical utility of the models. The results indicated that all five models provided good net benefits in predicting survival rates at different time points, with the RF models showing a slightly higher net benefit in the training group (**Figure 3**). Based on 1,000 bootstrap resamples, the RF models also exhibited consistent net benefits across a range of threshold probabilities (**Supplementary Table S3**). Calibration curves further demonstrated the good agreement between the predicted survival outcomes by the RF model and actual outcomes (**Figure 4**). The confusion matrix illustrated the performance of the RF classifier in the training and internal test groups (**Supplementary Figure S2**). Therefore, the RF models were identified as the optimal models for predicting prognosis in BCLM patients.

**Figure 5:**
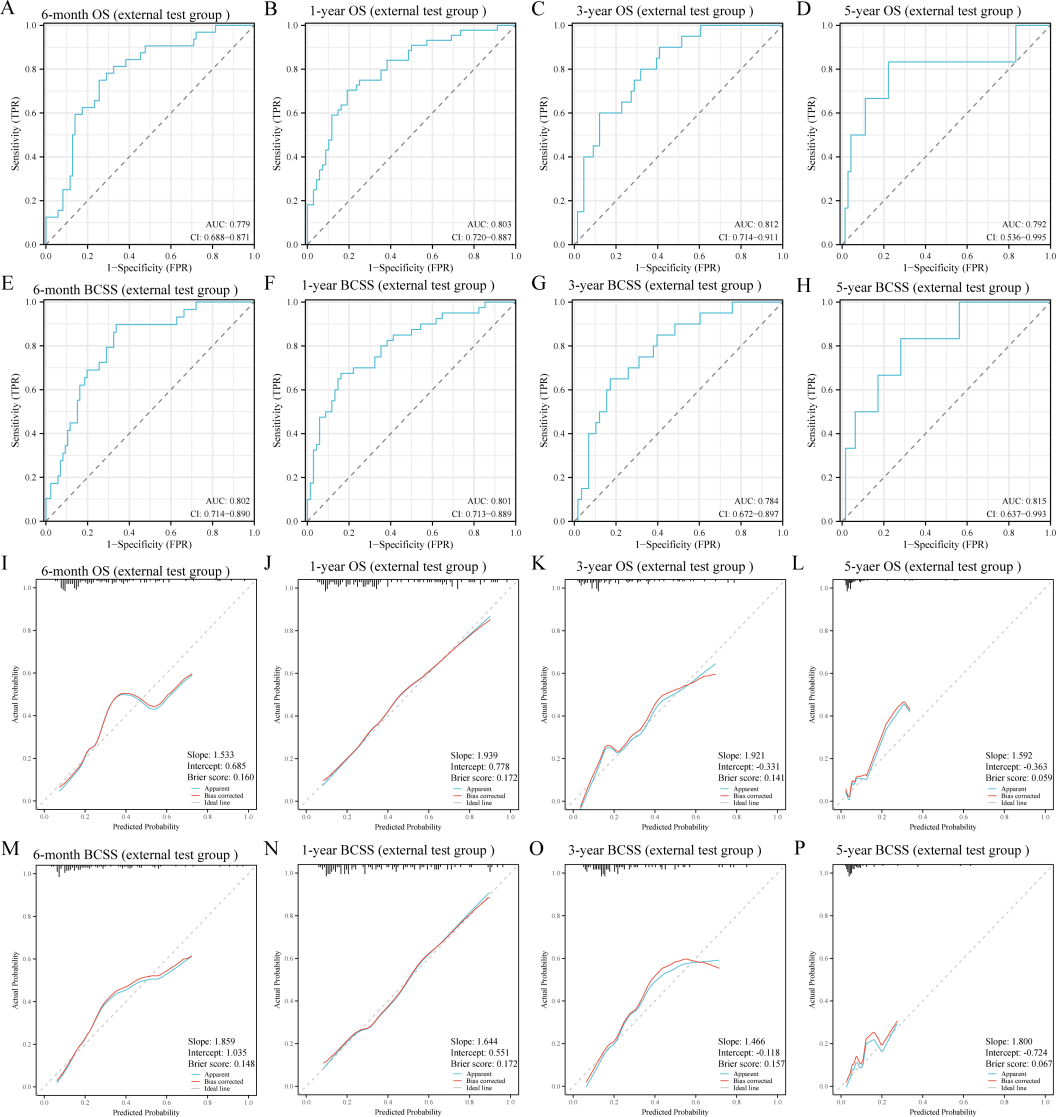
Receiver operating characteristic **(A-H)** and calibration curves **(I-P)** of the random forest models in the external test group. OS, overall survival; BCSS, breast cancer-specific survival; AUC, area under the curve.

**Figure 6:**
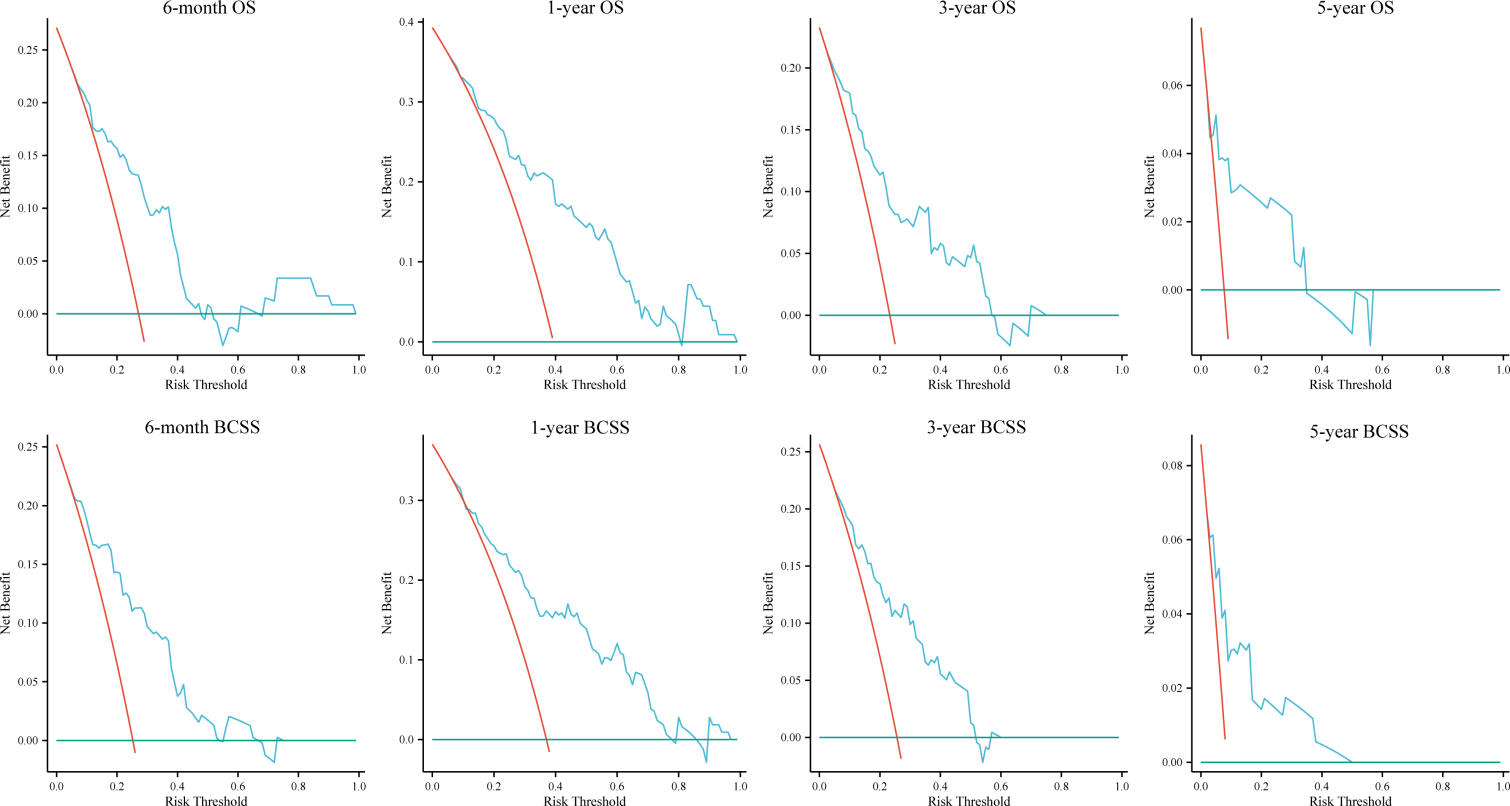
Decision curves for the random forest models in the external test group. OS, overall survival; BCSS, breast cancer-specific survival.

To further validate the robustness and applicability of the RF models, we included a total of 124 BCLM patients from the JCH and CHSU cohorts. The RF models demonstrated excellent performance in the external test cohort, as evidenced by their high discriminatory ability (**Figures 5A–H**). Specifically, the AUC values were as follows: 6-month OS, 0.779 (95% CI: 0.688–0.871); 1-year OS, 0.803 (95% CI:0.720–0.887); 3-year OS, 0.812 (95% CI: 0.714–0.911); 5-year OS, 0.792 (95% CI: 0.536–0.995); 6-month BCSS, 0.802 (95% CI: 0.714–0.890); 1-year BCSS, 0.801 (95% CI: 0.713–0.889); 3-year BCSS, 0.784 (95% CI: 0.672–0.897); and 5-year BCSS, 0.815 (95% CI: 0.637–0.993). Moreover, calibration curves further indicated good agreement between the predicted and actual outcomes (**Figures 5I–P**). DCA revealed that the RF models also exhibited good net benefit in the external cohort (**Figure 6**). Therefore, the RF models were considered the optimal tools for predicting the prognosis of BCLM patients.

### Sensitivity analysis

Since 38.20% of patients had unknown grade, we excluded these cases to validate the robustness of the RF models. The RF models were retrained with the same hyperparameters as the original model (**Supplementary Table S4**) and evaluated on the identical internal and external test groups. The retrained RF models achieved AUCs ranging from 0.858 to 0.899 in the training group, 0.741 to 0.831 in the internal test group, and 0.772 to 0.848 in the external test group (**Supplementary Figure S3**). Given that performance remained comparable, these findings suggested that including patients with unknown grade did not materially compromise the robustness of our RF models.

### Interpretability of the RF models

**Figure 7:**
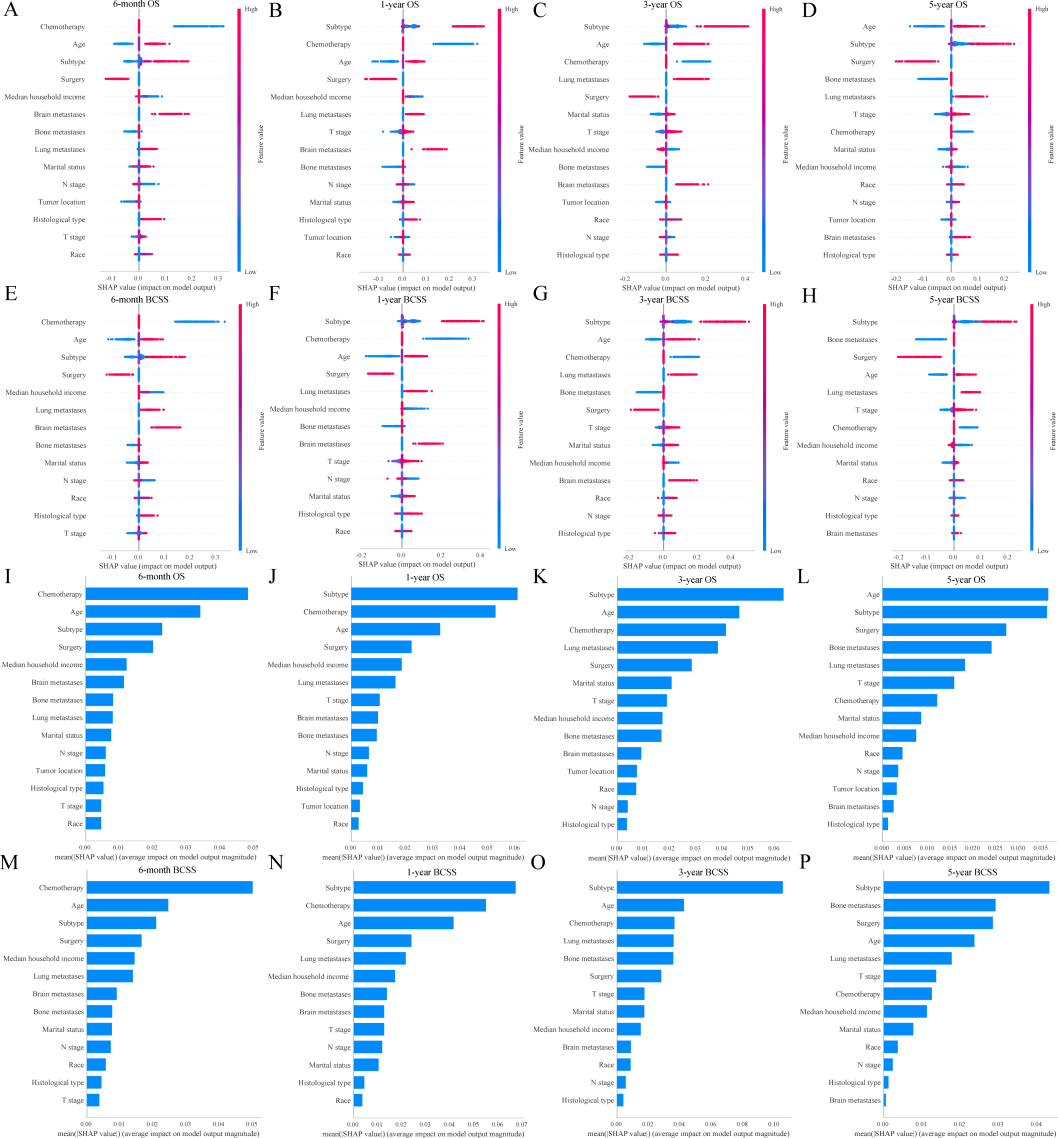
SHAP interprets the random forest models. SHAP values for each feature at different levels in the random forest models **(A-H)**; Importance of features in the random forest models **(I-P)**. OS, overall survival; BCSS, breast cancer-specific survival.

The SHAP analysis provided an explanation of how the RF models predicted survival outcomes and calculated the importance of features. **Figures 7A–H** showed the SHAP values for each feature at different levels. As the feature values increased, the redder the color became, and vice versa, the bluer the color was. Additionally, we ranked the features of the models (**Figures 7I–P**). The higher feature ranking indicated that the feature was more important, which meant that the feature contributed more to the model. Overall, chemotherapy, age, subtype, and surgery were the most valued by the RF models for predicting 6-month, 1-year, 3-year, and 5-year OS and BCSS. Additionally, two representative samples were provided for each model to elucidate the composition of the model outputs. Each feature’s weight was depicted in blue or red, indicative of its contribution toward a positive outcome. The value of each feature denoted its proportionate influence on the final score. **Supplementary Figures S4A–H** presented the detailed scores of surviving patients predicted to be alive, whereas **Supplementary Figures S4I–P** displayed the scores of dying patients predicted to be dead.

### Prognostic impact of PTS in BCLM patients

**Figure 8:**
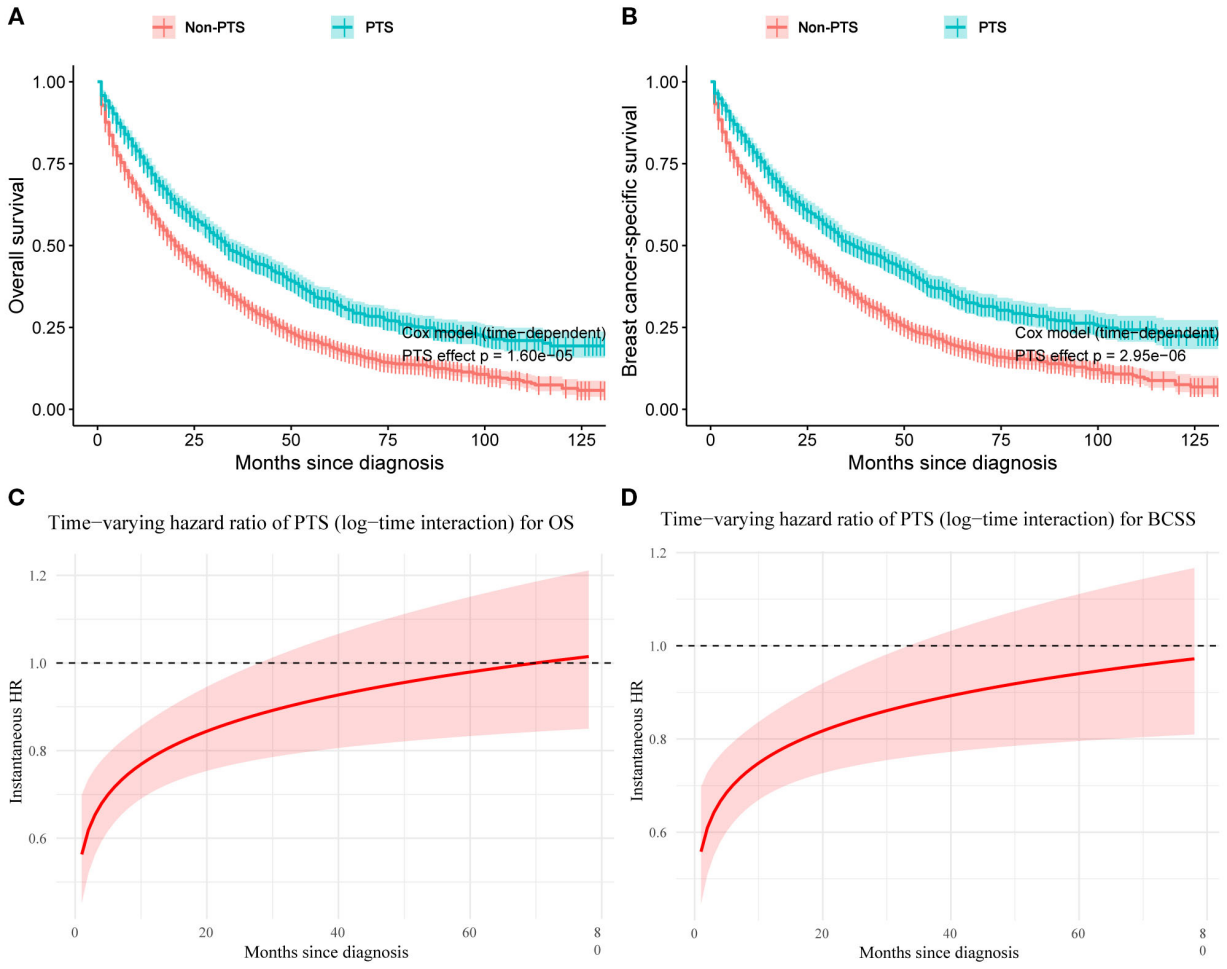
Simon–Makuch plots with primary tumor surgery as a time-dependent variable for overall survival **(A)** and breast cancer–specific survival **(B)**. Time-varying hazard ratio curves for the effect of primary tumor surgery on overall survival **(C)** and breast cancer–specific survival **(D)**. PTS, primary tumor surgery; OS, overall survival; BCSS, breast cancer-specific survival.

Baseline characteristics of the PTS and non-PTS groups were shown in **Supplementary Table S5**. The adjusted time-dependent Cox model demonstrated a reduced risk of OS events in the PTS group (HR=0.80, 95% CI: 0.72–0.88; P< 0.001; **Table 4**). Similarly, BCSS risk was also lower (HR=0.77, 95% CI: 0.69–0.86; P< 0.001). The Simon–Makuch curves illustrated survival differences over time between the PTS and non-PTS groups (**Figures 8A, B**). As shown in **Figures 8C, D**, even after accounting for time-varying effects, the instantaneous HRs for both OS and BCSS remained consistently< 1 across the majority of the follow-up period.

**Table 4:** Time-dependent Cox analyses of patients with breast cancer liver metastases in the SEER database.

Covariates	OS		BCSS	
	HR (95% CI)	P	HR (95% CI)	P
PTS (time-varying)	0.80 (0.72–0.88)	<0.001	0.77 (0.69–0.86)	<0.001
Age				
≤50	Reference		Reference	
51–65	1.30 (1.19–1.42)	<0.001	1.31 (1.19–1.43)	<0.001
≥66	1.83 (1.66–2.02)	<0.001	1.77 (1.59–1.96)	<0.001
Race				
White	Reference		Reference	
Black	1.27 (1.15–1.39)	<0.001	1.25 (1.13–1.38)	<0.001
Others	0.94 (0.83–1.08)	0.384	0.93 (0.81–1.07)	0.321
Marital status				
Married	Reference		Reference	
Single/homosexual	1.07 (0.97–1.17)	0.158	1.06 (0.96–1.17)	0.229
Widow/divorced/others	1.20 (1.10–1.31)	<0.001	1.20 (1.10–1.31)	<0.001
Median household income				
<40,000	Reference		Reference	
40,000–59,999	0.97 (0.80–1.17)	0.74	1.02 (0.83–1.25)	0.864
60,000–69,999	0.85 (0.70–1.03)	0.094	0.90 (0.73–1.11)	0.317
70,000+	0.72 (0.60–0.87)	<0.001	0.77 (0.63–0.94)	0.009
Histological type				
Invasive ductal carcinoma	Reference		Reference	
Invasive lobular carcinoma	1.11 (0.95–1.30)	0.175	1.10 (0.94–1.29)	0.247
Mixed	1.03 (0.87–1.21)	0.751	1.01 (0.85–1.20)	0.887
Other	1.12 (0.99–1.26)	0.069	1.14 (1.01–1.29)	0.038
Tumor location				
Upper outer	Reference		Reference	
Lower outer	1.16 (0.98–1.38)	0.089	1.15 (0.96–1.38)	0.119
Lower inner	0.93 (0.75–1.14)	0.464	0.94 (0.76–1.16)	0.565
Upper inner	1.03 (0.88–1.21)	0.715	1.01 (0.86–1.20)	0.864
Central	1.08 (0.92–1.27)	0.334	1.11 (0.94–1.30)	0.232
Others	1.12 (1.03–1.23)	0.009	1.12 (1.02–1.22)	0.017
Grade				
G1	Reference		Reference	
G2	1.17 (0.94–1.46)	0.162	1.19 (0.95–1.50)	0.134
G3	1.56 (1.25–1.95)	<0.001	1.62 (1.29–2.04)	<0.001
Unknown	1.42 (1.14–1.78)	0.002	1.43 (1.14–1.81)	0.002
Subtype				
HR+/HER2-	Reference		Reference	
HR+/HER2+	0.61 (0.55–0.68)	<0.001	0.61 (0.55–0.68)	<0.001
HR-/HER2+	0.75 (0.67–0.83)	<0.001	0.75 (0.66–0.84)	<0.001
HR-/HER2-	2.12 (1.91–2.35)	<0.001	2.14 (1.92–2.38)	<0.001
T stage				
T1	Reference		Reference	
T2	1.10 (0.97–1.25)	0.137	1.10 (0.96–1.25)	0.157
T3	1.13 (0.98–1.30)	0.096	1.14 (0.98–1.31)	0.085
T4	1.29 (1.13–1.47)	<0.001	1.29 (1.13–1.48)	<0.001
N stage				
N0	Reference		Reference	
N1	0.83 (0.76–0.91)	<0.001	0.82 (0.74–0.90)	<0.001
N2	0.87 (0.77–1.00)	0.046	0.85 (0.74–0.97)	0.019
N3	0.83 (0.74–0.94)	0.004	0.83 (0.73–0.94)	0.004
Bone metastases				
No/unknown	Reference		Reference	
Yes	1.28 (1.18–1.38)	<0.001	1.31 (1.21–1.42)	<0.001
Brain metastases				
No/unknown	Reference		Reference	
Yes	1.59 (1.41–1.79)	<0.001	1.58 (1.40–1.78)	<0.001
Lung metastases				
No/unknown	Reference		Reference	
Yes	1.29 (1.19–1.39)	<0.001	1.30 (1.20–1.41)	<0.001
Systemic therapy before surgery				
No	Reference		Reference	
Yes	0.67 (0.57–0.78)	<0.001	0.68 (0.57–0.80)	<0.001

OS, overall survival; BCSS, breast cancer-specific survival; HR, hazard ratio; CI, confidence interval.

The piecewise Cox model further supported these findings: for OS, the HR was 0.83 (95% CI: 0.75–0.92) within the first 24 months and 0.73 (95% CI: 0.61–0.88) thereafter; for BCSS, the HR was 0.80 (95% CI: 0.72–0.89) within 0–24 months and 0.68 (95% CI: 0.56–0.83) beyond 24 months (**Supplementary Table S6**). The 2-month landmark analysis similarly showed that patients receiving PTS after systemic therapy had improved OS (HR=0.60, 95% CI: 0.37–0.97; p = 0.038) and BCSS (HR=0.60, 95% CI: 0.36–0.98; p = 0.043), consistent with the primary model (**Supplementary Table S7**). We further quantified the robustness of our findings using the E-value. For OS and BCSS, the E-values were 1.81 and 1.92, respectively (lower bound E-values: OS=1.53; BCSS=1.60). This suggested that to nullify the observed survival benefits of PTS, an unmeasured confounder would need to be associated with both PTS treatment and the outcomes with an HR ≥ 1.81 or 1.92. Similarly, in the 2-month landmark analysis, the E-value for OS (HR=0.60) was 2.72 (lower bound: 1.21), and for BCSS (HR=0.60) was 2.72 (lower bound: 1.16). These results indicated that only an unmeasured confounder with at least moderate strength could substantially alter our conclusions. In summary, our findings confirm the survival benefit of PTS in improving both OS and BCSS.

## Discussion

Although the prevalence of BC screening has led to a year-by-year decrease in the proportion of newly diagnosed patients with advanced-stage BC, approximately 6%–7% of newly diagnosed BC patients still present with metastatic lesions at the initial diagnosis (27). Patients with liver-only metastasis are relatively rare, accounting for 5%–12% of cases (3). Individuals with BCLM generally exhibit weaker physical and nutritional conditions. This vulnerability heightens the risk of encountering complications during treatment, including liver failure, jaundice, continuous ascites, portal vein thrombosis, and cachexia, potentially leading to premature mortality (28). Furthermore, most chemotherapeutic and endocrine agents, such as taxanes, fluoropyrimidines, aromatase inhibitors, and CDK4/6 inhibitors, are metabolized by the liver, thereby increasing its workload. Consequently, it is imperative to comprehensively consider the status of metastatic organs, overall nutritional condition, anticipated survival, as well as the efficacy and safety of the treatment in the treatment decision-making for BCLM patients. This study aimed to develop novel predictive models for OS and BCSS in BCLM patients using ML algorithms, facilitating the formulation of effective treatment strategies. To our knowledge, this was the largest study to date analyzing prognosis and PTS in BCLM patients. For the first time, we had developed OS and BCSS prediction models based on five different ML algorithms. Our RF models demonstrated outstanding accuracy in predicting 6-month, 1-year, 3-year, and 5-year OS and BCSS for BCLM patients.

Our study identified several independent risk factors significantly associated with OS and BCSS, including older age, being African American, being divorced, having other pathological types, HR-/HER2- status, stage T4, and the presence of additional distant metastases. Conversely, protective factors included a household income exceeding $60,000, HR+/HER2+ status, stage N1, and the administration of surgery and chemotherapy. Recent studies have corroborated that older age correlates with poorer OS and BCSS (8, 29, 30). Our findings revealed that HR+/HER2+ patients exhibited the most favorable prognosis, whereas those with HR-/HER2- had poorer outcomes. Historically, luminal-type BC was deemed to have a relatively favorable prognosis. However, with the widespread use of HER2-targeted therapies, similar survival rates have been observed among patients with advanced BC in the HR+/HER2- and HR-/HER2+ subtypes. For HR+ patients, the combination of CDK4/6 inhibitors and aromatase inhibitors as first-line treatment significantly enhanced progression-free survival (PFS) in advanced cases (31, 32). Data from the PHOEBE trial demonstrated that for HER2+ patients with visceral metastases, pyrrolitinib combined with capecitabine extended PFS to 12.5 months, compared to lapatinib combined with capecitabine in those previously treated with trastuzumab and paclitaxel (33). Furthermore, consistent with other studies findings, chemotherapy has been shown to significantly improve patient prognosis (8, 34). The T4 stage indicates locally advanced disease, reflecting the extent of primary tumor invasion and burden, and is often associated with a poorer prognosis. Unlike oligometastatic disease, the presence of concurrent metastases to other organs in BCLM patients is another critical factor leading to adverse outcomes (35, 36).

In our comparative analysis of five ML models, the RF algorithm emerged as the best-performing model. It outperformed all other models in the large American cohort and was consistently validated in cross-national cohorts. Chen et al. constructed a nomogram to predict OS in BCLM patients, which achieved an internal concordance index (C-index) of 0.685 (29). A recent study built a traditional nomogram prediction model for young BCLM patients using a single SEER dataset (34), which showed AUCs of 0.753, 0.773, and 0.761 for predicting 1-year, 2-year, and 5-year OS, respectively, and 0.755, 0.781, and 0.787 for 1-year, 2-year, and 5-year BCSS, respectively. In contrast, our RF model demonstrated superior predictive performance, with AUCs ranging from 0.840 to 0.889 for predicting OS at different time points in the training group, and 0.763 to 0.775 for the internal test group. Furthermore, the RF model showed AUCs ranging from 0.849 to 0.867 for predicting BCSS at different time points in the training group, and 0.765 to 0.787 in the internal test group. Importantly, our model was validated using independent cohorts from two hospitals in China, with AUC values ranging from 0.779 to 0.815. To probe robustness to missingness in key clinicopathologic variables, we performed a sensitivity analysis that excluded patients with unknown grade and retrained the RF models with the same hyperparameters; the models maintained comparable discrimination across training, internal, and external cohorts (AUCs ranged 0.858–0.899, 0.741–0.831, and 0.772–0.848, respectively), indicating that our findings are not materially drivenxby grade-related missingness. Collectively, these results underscore the stability and generalizability of the RF framework across populations and analytic specifications. The enhanced predictive performance provided more reliable evidence for clinicians in managing and stratifying BCLM patients. Meanwhile, DCA confirmed that our RF model demonstrated excellent clinical utility.

Studies have shown that PTS in advanced BC can improve local symptoms, significantly prolong progression free survival (PFS), and enhance quality of life (37–39). However, there remains controversy over whether PTS can extend OS. The TATA study in India found that in patients with stage IV BC who responded to first-line therapy, PTS did not provide an OS benefit (23). Similarly, the EA2108 study, after 4–8 months of systemic treatment, performed PTS in patients with effective systemic therapy but did not extend OS, although it did improve PFS (39). However, no definitive conclusion has been reached on whether PTS should be performed in BCLM patients. In our study, to reduce selection bias and, critically, to minimize immortal-time bias, we modeled PTS as a time-dependent exposure and applied a suite of complementary analyses, including time-dependent Cox models with time-varying effects, Simon-Makuch curves, a segmented Cox model, and a 2-month landmark analysis. Across these approaches, PTS was consistently associated with lower risks of death and BC–specific mortality (OS: HR=0.80, 95% CI 0.72–0.88; BCSS: HR=0.77, 95% CI 0.69–0.86), with hazard ratios remaining<1 for most of follow-up. Effect sizes were comparable in piecewise analyses (e.g., OS HR=0.83 within 24 months and 0.73 beyond 24 months; BCSS HR=0.80 and 0.68, respectively) and persisted in the landmark cohort (OS HR=0.60; BCSS HR=0.60). The SEER database lacks information on endocrine and targeted therapies, both of which are established prognostic factors in BCLM; their absence may introduce residual confounding. E-values (OS 1.81; BCSS 1.92; landmark OS/BCSS 2.72) indicate that only an unmeasured confounder of at least moderate strength could negate these associations, supporting the robustness of our findings. While causality cannot be definitively established in an observational setting, our results are consistent with longer survival being associated with PTS among carefully selected BCLM patients—particularly those managed with contemporary systemic therapy—supporting careful consideration of individualized, multidisciplinary evaluation rather than a blanket proscription or endorsement.

Despite the promising results, there are several limitations to our study. First, the SEER database lacks granular treatment details (e.g., endocrine and targeted therapies) and progression/recurrence data, which may limit predictive precision. Although time-dependent Cox modeling, landmark analyses, and E-value calculations were used to mitigate immortal-time bias and test robustness, unmeasured factors may still affect the observed PTS associations. Additionally, missing grade information—despite supportive sensitivity analyses—could introduce residual bias. Furthermore, limited sample sizes in certain subgroups precluded stratified analyses, thereby restricting assessment of model stability across subsets. Finally, our external validation cohorts were relatively small and regionally restricted, underscoring the need for larger multicenter studies.

## Conclusion

In conclusion, we developed and externally validated robust RF-based models for predicting OS and BCSS in BCLM patients. Our results indicate that PTS is associated with longer survival and lower breast cancer-specific mortality in carefully selected patients, supporting consideration within individualized, multidisciplinary decision-making.

## Data Availability

The raw data supporting the conclusions of this article will be made available by the authors, without undue reservation.

## Glossary

AUC: area under the curve
BC: breast cancer
BCSS: breast cancer-specific survival
BCLM: breast cancer liver metastases
CHSU: Cancer Hospital of Shantou University Medical College
CI: confidence interval
DCA: decision curve analysis
DT: decision tree
HER2: human epidermal growth factor receptor 2
GBDT: gradient boosting decision tree
GVIF: generalized variance inflation factor
HR: hazard ratio
ICD-O-3: International Classification of Diseases for Oncology, 3rd Edition
IDC: invasive ductal carcinoma
IPCW: inverse probability-of-censoring weighting
JCH: Jiangmen Central Hospital
LR: logistic regression
ML: machine learning
OS: overall survival
PTS: primary tumor surgery
RF: Random Forest
SEER: Surveillance, Epidemiology, and End Results
SHAP: SHAPley Additive exPlanations
XGBoost: extreme gradient boosting
