# Supplementary material for: Machine learning algorithms for individualized prediction of prognosis in breast cancer liver metastases and the prognostic impact of primary tumor surgery: a multicenter study
Source: Front Endocrinol (Lausanne). 2025 Oct 13;16:1656191. doi: 10.3389/fendo.2025.1656191 (PMC12554575; doi:10.3389/fendo.2025.1656191)
Supplement: Supplementary file 1 [file SupplementaryFile1.docx]

**Machine learning algorithms for individualised prediction of prognosis in breast cancer liver metastases and the prognostic impact of primary tumor surgery: a multicenter study**

Chunmei Chen^1^, Jundong Wu^2^, Bo Xu^3,4^, Weiwen Li^1^, Chengming Zhong^5^, Zhibing Yan^6^, Qipeng Zhong^6^, Ronggang Li^7^, Mingtao Shao^1^, Yan Dong^1^, Yutong Fang^2^,Yong Li^1*^and Qunchen Zhang^1*^

1. Department of Breast, Jiangmen Central Hospital, Jiangmen, Guangdong 529030, P.R. China.
2. The Breast Center, Cancer Hospital of Shantou University Medical College, Shantou, Guangdong 515041, P.R. China.
3. Department of General Surgery, The First Affiliated Hospital of Jinan University, Guangzhou, Guangdong 510000 P.R.China.
4. Department of General Surgery, Guangzhou First People’s Hospital, School of Medicine, South China University of Technology, Guangzhou,Guangdong 510000 P.R.China.
5. Department of Equipment, Jiangmen Xinhui Maternal and Child Health Hospital, Jiangmen, Guangdong 529030, P.R. China.
6. Department of Anesthesiology, Jiangmen Central Hospital, Jiangmen, Guangdong 529030, P.R. China.
7. Department of pathology, Jiangmen Central Hospital, Jiangmen, Guangdong 529030, P.R. China.

* These authors contributed equally to this work.


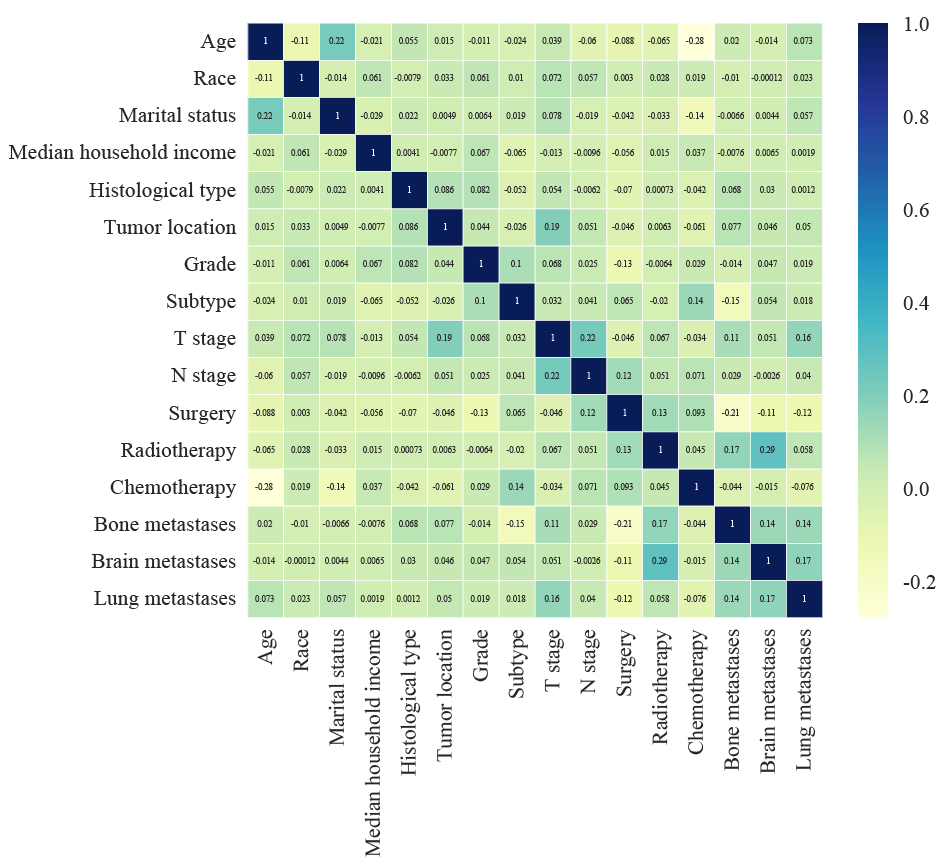


Supplementary Figure.1 Correlation between clinical characteristic data.


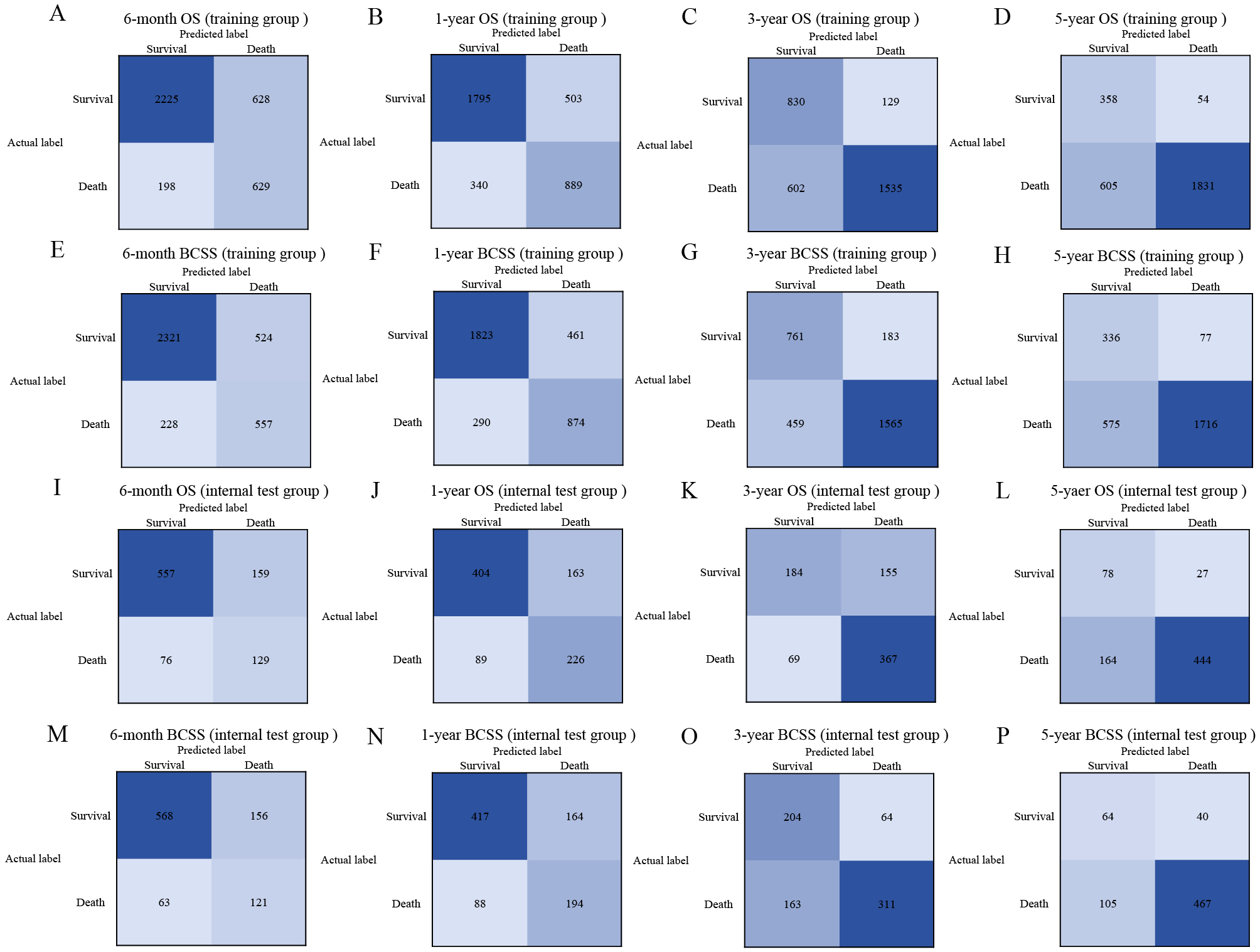


Supplementary Figure.2 Confusion matrix of the random forest models' predicted results in the training group (**A-H**) and internal test group (**I-P**). OS, overall survival; BCSS, breast cancer-specific survival.


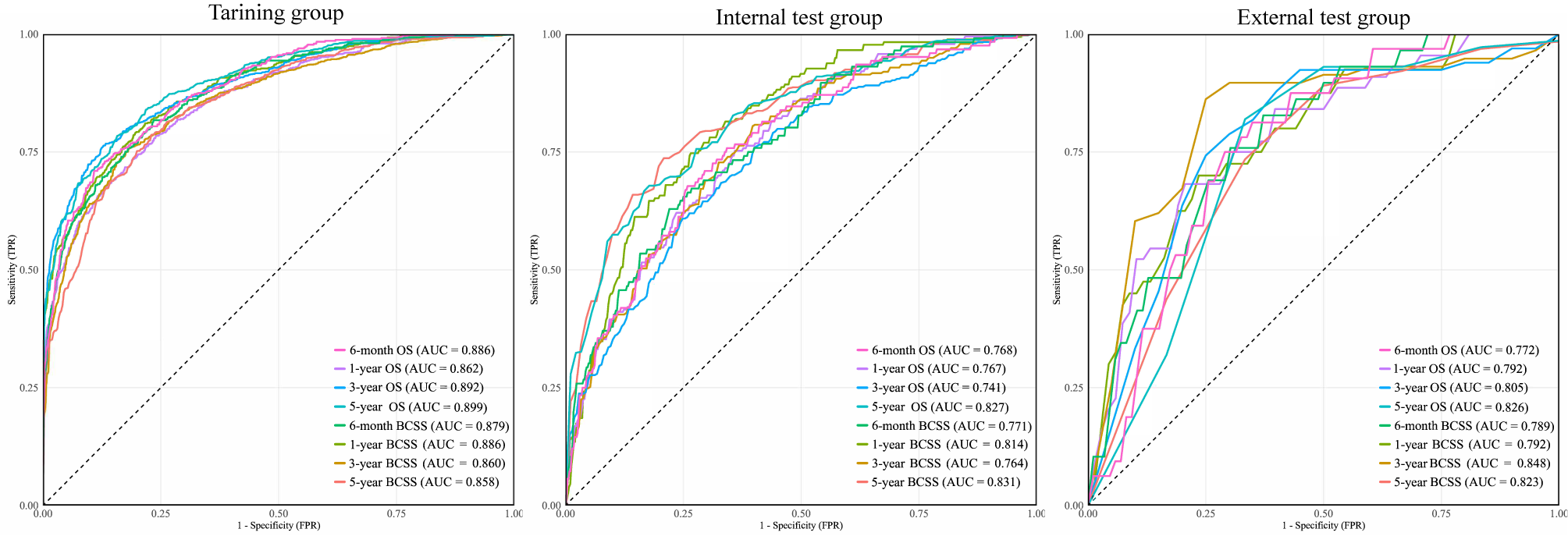


Supplementary Figure.3 Receiver operating characteristic curves of the retrained random forest models in the training, internal test, and external test cohorts. OS, overall survival; BCSS, breast cancer-specific survival.


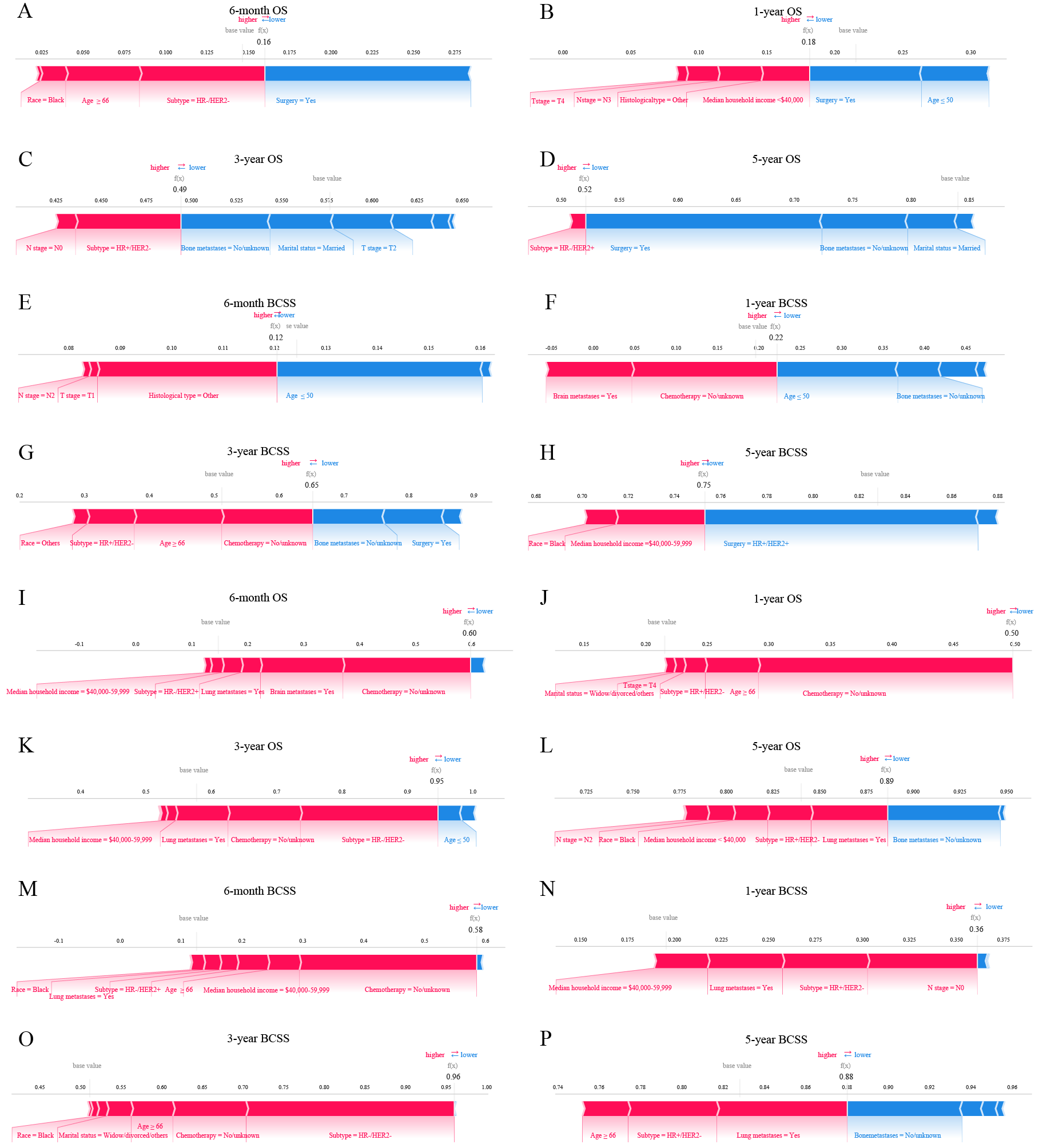


Supplementary Figure.4 Composition of scores for actual survivors predicted as survivors (**A-H**); Composition of scores for actual deceased patients predicted as deceased (**I-P**). OS, overall survival; BCSS, breast cancer-specific survival.

Table S1. Survival outcomes in the SEER and external cohorts

| Timepoint | SEER cohort | |  | External cohort | |
| --- | --- | --- | --- | --- | --- |
|  | Survival rate | Events |  | Survival rate | Events |
| 6-month OS | 78.20% | 1032 |  | 74.00% | 32 |
| 1-year OS | 66.60% | 1544 |  | 63.30% | 44 |
| 3-year OS | 37.30% | 2659 |  | 37.20% | 66 |
| 5-year OS | 23.40% | 3044 |  | 24.10% | 72 |
| 6-month BCSS | 79.40% | 969 |  | 76.00% | 29 |
| 1-year BCSS | 68.40% | 1446 |  | 65.90% | 40 |
| 3-year BCSS | 39.60% | 2498 |  | 43.90% | 58 |
| 5-year BCSS | 25.50% | 2863 |  | 28.50% | 64 |

Abbreviations: SEER, Surveillance, Epidemiology, and End Results; OS, overall survival; BCSS, breast cancer-specific survival

Table S2. Analysis of multicollinearity between variables

| Variables | GVIF | DF | GVIF^(1/(2\*DF)) |
| --- | --- | --- | --- |
| Age | 1.237 | 2 | 1.055 |
| Race | 1.130 | 2 | 1.031 |
| Marital status | 1.176 | 2 | 1.041 |
| Median household income (inflation adjusted) | 1.067 | 3 | 1.011 |
| Histological type | 1.112 | 3 | 1.018 |
| Tumor location | 1.107 | 5 | 1.010 |
| Subtype | 1.146 | 3 | 1.023 |
| T stage | 1.203 | 3 | 1.031 |
| N stage | 1.143 | 3 | 1.022 |
| Surgery | 1.125 | 1 | 1.061 |
| Chemotherapy | 1.151 | 1 | 1.073 |
| Bone metastases | 1.122 | 1 | 1.059 |
| Brain metastases | 1.064 | 1 | 1.032 |
| Lung metastases | 1.105 | 1 | 1.051 |

Abbreviations: GVIF, generalized variance inflation factor; DF, degrees of freedom.

Table S3. Net benefit of the random forest models at different threshold probabilities

| Endpoint | Threshold | Internal test group | |  | External test group | |
| --- | --- | --- | --- | --- | --- | --- |
|  |  | Net benefit | 95%CI |  | Net benefit | 95%CI |
| 6-month OS | 0.10 | 0.13 | 0.10–0.16 |  | 0.18 | 0.09–0.27 |
|  | 0.20 | 0.09 | 0.06–0.11 |  | 0.15 | 0.07–0.24 |
|  | 0.30 | 0.05 | 0.03–0.08 |  | 0.10 | 0.03–0.17 |
| 1-year OS | 0.20 | 0.20 | 0.16–0.23 |  | 0.24 | 0.13–0.34 |
|  | 0.30 | 0.14 | 0.11–0.17 |  | 0.20 | 0.10–0.30 |
|  | 0.40 | 0.10 | 0.06–0.13 |  | 0.17 | 0.08–0.27 |
| 3-year OS | 0.40 | 0.42 | 0.37–0.47 |  | 0.57 | 0.42–0.72 |
|  | 0.50 | 0.35 | 0.29–0.41 |  | 0.47 | 0.30–0.65 |
|  | 0.60 | 0.29 | 0.23–0.35 |  | 0.45 | 0.26–0.63 |
| 5-year OS | 0.50 | 0.70 | 0.65–0.75 |  | 0.83 | 0.68–0.95 |
|  | 0.70 | 0.55 | 0.47–0.62 |  | 0.75 | 0.55–0.92 |
|  | 0.80 | 0.44 | 0.34–0.53 |  | 0.64 | 0.36–0.86 |
| 6-month BCSS | 0.10 | 0.15 | 0.12–0.18 |  | 0.20 | 0.11–0.29 |
|  | 0.20 | 0.10 | 0.07–0.12 |  | 0.15 | 0.07–0.24 |
|  | 0.30 | 0.06 | 0.03–0.08 |  | 0.10 | 0.03–0.18 |
| 1-year BCSS | 0.20 | 0.23 | 0.19–0.27 |  | 0.27 | 0.15–0.37 |
|  | 0.30 | 0.17 | 0.13–0.20 |  | 0.22 | 0.12–0.32 |
|  | 0.40 | 0.13 | 0.09–0.16 |  | 0.18 | 0.08–0.27 |
| 3-year BCSS | 0.40 | 0.48 | 0.42–0.53 |  | 0.61 | 0.46–0.75 |
|  | 0.50 | 0.40 | 0.34–0.45 |  | 0.53 | 0.37–0.70 |
|  | 0.60 | 0.32 | 0.26–0.38 |  | 0.49 | 0.31–0.67 |
| 5-year BCSS | 0.50 | 0.71 | 0.66–0.76 |  | 0.85 | 0.71–0.95 |
|  | 0.70 | 0.60 | 0.53–0.67 |  | 0.79 | 0.62–0.93 |
|  | 0.80 | 0.46 | 0.36–0.55 |  | 0.69 | 0.44–0.89 |

Abbreviations: OS, overall survival; BCSS, breast cancer-specific survival; CI, confidence interval

Table S4. Optimal random forest hyperparameter combinations for overall survival and breast cancer-specific survival at different time points

| Parameters | n estimators | max depth | min samples split | min samples leaf |
| --- | --- | --- | --- | --- |
| 6-month OS | 100 | 7 | 2 | 1 |
| 1-year OS | 100 | 7 | 2 | 1 |
| 3-year OS | 90 | 8 | 2 | 1 |
| 5-year OS | 100 | 7 | 2 | 1 |
| 6-month BCSS | 100 | 7 | 2 | 1 |
| 1-year BCSS | 80 | 8 | 2 | 1 |
| 3-year BCSS | 90 | 8 | 2 | 1 |
| 5-year BCSS | 100 | 6 | 2 | 1 |

Abbreviations: OS, overall survival; BCSS, breast cancer-specific survival

Table S5. Baseline characteristics of patients with breast cancer liver metastases

| Variables | | Non-PTS | PTS |
| --- | --- | --- | --- |
| Age |  |  |  |
|  | ≤50 | 1099 (30.0) | 335 (35.1) |
|  | 51–65 | 1494 (40.8) | 384 (40.2) |
|  | ≥66 | 1072 (29.2) | 236 (24.7) |
| Race |  |  |  |
|  | White | 2650 (72.3) | 702 (73.5) |
|  | Black | 647 (17.7) | 158 (16.5) |
|  | Others | 368 (10.0) | 95 (9.9) |
| Marital status |  |  |  |
|  | Married | 1635 (44.6) | 461 (48.3) |
|  | Single/homosexual | 918 (25.0) | 226 (23.7) |
|  | Widow/divorced/others | 1112 (30.3) | 268 (28.1) |
| Median household income |  |  |  |
|  | <$40,000 | 114 (3.1) | 37 (3.9) |
|  | $40,000-59,999 | 761 (20.8) | 233 (24.4) |
|  | $60,000-69,999 | 736 (20.1) | 219 (22.9) |
|  | $70,000+ | 2054 (56.0) | 466 (48.8) |
| Histological type |  |  |  |
|  | IDC | 2865 (78.2) | 813 (85.1) |
|  | ILC | 239 (6.5) | 36 (3.8) |
|  | Mixed | 161 (4.4) | 61 (6.4) |
|  | Other | 400 (10.9) | 45 (4.7) |
| Tumor location |  |  |  |
|  | Upper outer | 916 (25.0) | 270 (28.3) |
|  | Lower outer | 183 (5.0) | 64 (6.7) |
|  | Lower inner | 131 (3.6) | 40 (4.2) |
|  | Upper inner | 235 (6.4) | 63 (6.6) |
|  | Central | 220 (6.0) | 53 (5.5) |
|  | Others | 1980 (54.0) | 465 (48.7) |
| Grade |  |  |  |
|  | G1 | 93 (2.5) | 22 (2.3) |
|  | G2 | 819 (22.3) | 226 (23.7) |
|  | G3 | 1145 (31.2) | 538 (56.3) |
|  | Unknown | 1608 (43.9) | 169 (17.7) |
| Subtype |  |  |  |
|  | HR+/HER2- | 1682 (45.9) | 379 (39.7) |
|  | HR+/HER2+ | 875 (23.9) | 226 (23.7) |
|  | HR-/HER2+ | 583 (15.9) | 170 (17.8) |
|  | HR-/HER2- | 525 (14.3) | 180 (18.8) |
| T stage |  |  |  |
|  | T1 | 402 (11.0) | 118 (12.4) |
|  | T2 | 1234 (33.7) | 372 (39.0) |
|  | T3 | 660 (18.0) | 187 (19.6) |
|  | T4 | 1369 (37.4) | 278 (29.1) |
| N stage |  |  |  |
|  | N0 | 741 (20.2) | 169 (17.7) |
|  | N1 | 2072 (56.5) | 391 (40.9) |
|  | N2 | 343 (9.4) | 210 (22.0) |
|  | N3 | 509 (13.9) | 185 (19.4) |
| Bone metastases |  |  |  |
|  | No/unknown | 1273 (34.7) | 550 (57.6) |
|  | Yes | 2392 (65.3) | 405 (42.4) |
| Brain metastases |  |  |  |
|  | No/unknown | 3273 (89.3) | 923 (96.6) |
|  | Yes | 392 (10.7) | 32 (3.4) |
| Lung metastases |  |  |  |
|  | No/unknown | 2352 (64.2) | 726 (76.0) |
|  | Yes | 1313 (35.8) | 229 (24.0) |
| Systemic therapy before surgery |  |  |  |
|  | No | 3665 (100.0) | 619 (64.8) |
|  | Yes | 0 (0.0) | 336 (35.2) |

Abbreviations: PTS, primary tumor surgery; IDC, invasive ductal carcinoma; ILC, invasive lobular carcinoma

Table S6. Piecewise Cox analyses of patients with breast cancer liver metastases in the SEER database

| Interval | Covariate | OS | |  | BCSS | |
| --- | --- | --- | --- | --- | --- | --- |
|  |  | HR (95% CI) | P |  | HR (95% CI) | P |
| 0–24 months | PTS (time-varying) | 0.83 (0.75–0.92) | ＜0.001 |  | 0.80 (0.72–0.89) | ＜0.001 |
| >24 months | PTS (time-varying) | 0.73 (0.61–0.88) | 0.001 |  | 0.68 (0.56–0.83) | ＜0.001 |

Abbreviations: SEER, Surveillance, Epidemiology, and End Results; OS, overall survival; BCSS, breast cancer-specific survival; CI, confidence interval; PTS, primary tumor surgery; HR, hazard ratio; CI, confidence interval

Table S7. 2-month Landmark Cox analyses for patients with breast cancer liver metastases

| Covariates | OS | |  | BCSS | |
| --- | --- | --- | --- | --- | --- |
|  | HR (95% CI) | P |  | HR (95% CI) | P |
| Non-PTS | Reference |  |  | Reference |  |
| PTS after ≥2 months | 0.60 (0.37–0.97) | 0.038 |  | 0.60 (0.36–0.98) | 0.043 |
| Age |  |  |  |  |  |
| ≤50 | Reference |  |  | Reference |  |
| 51–65 | 1.29 (1.17–1.44) | <0.001 |  | 1.30 (1.17–1.45) | <0.001 |
| ≥66 | 1.66 (1.48–1.87) | <0.001 |  | 1.61 (1.43–1.82) | <0.001 |
| Race |  |  |  |  |  |
| White | Reference |  |  | Reference |  |
| Black | 1.30 (1.16–1.45) | <0.001 |  | 1.28 (1.14–1.44) | <0.001 |
| Others | 1.00 (0.86–1.16) | 0.979 |  | 0.99 (0.85–1.16) | 0.904 |
| Marital status |  |  |  |  |  |
| Married | Reference |  |  | Reference |  |
| Single/homosexual | 1.08 (0.97–1.20) | 0.17 |  | 1.07 (0.96–1.20) | 0.209 |
| Widow/divorced/others | 1.16 (1.05–1.29) | 0.003 |  | 1.15 (1.04–1.27) | 0.009 |
| Median household income |  |  |  |  |  |
| <40,000 | Reference |  |  | Reference |  |
| 40,000–59,999 | 0.90 (0.71–1.13) | 0.372 |  | 0.94 (0.74–1.20) | 0.604 |
| 60,000–69,999 | 0.80 (0.63–1.01) | 0.06 |  | 0.84 (0.66–1.07) | 0.161 |
| 70,000+ | 0.68 (0.54–0.85) | 0.001 |  | 0.71 (0.56–0.90) | 0.005 |
| Histological type |  |  |  |  |  |
| Invasive ductal carcinoma | Reference |  |  | Reference |  |
| Invasive lobular carcinoma | 1.14 (0.96–1.36) | 0.137 |  | 1.15 (0.96–1.38) | 0.125 |
| Mixed | 0.94 (0.77–1.15) | 0.554 |  | 0.94 (0.76–1.15) | 0.53 |
| Other | 1.09 (0.95–1.25) | 0.211 |  | 1.11 (0.97–1.28) | 0.132 |
| Tumor location |  |  |  |  |  |
| Upper outer | Reference |  |  | Reference |  |
| Lower outer | 1.09 (0.88–1.35) | 0.41 |  | 1.09 (0.87–1.36) | 0.449 |
| Lower inner | 0.93 (0.74–1.18) | 0.561 |  | 0.95 (0.75–1.21) | 0.677 |
| Upper inner | 0.97 (0.80–1.17) | 0.716 |  | 0.96 (0.79–1.17) | 0.708 |
| Central | 1.02 (0.85–1.23) | 0.824 |  | 1.04 (0.86–1.26) | 0.709 |
| Others | 1.08 (0.98–1.20) | 0.139 |  | 1.09 (0.98–1.21) | 0.115 |
| Grade |  |  |  |  |  |
| G1 | Reference |  |  | Reference |  |
| G2 | 1.08 (0.85–1.37) | 0.55 |  | 1.10 (0.86–1.42) | 0.454 |
| G3 | 1.41 (1.10–1.80) | 0.006 |  | 1.45 (1.12–1.87) | 0.004 |
| Unknown | 1.32 (1.03–1.69) | 0.026 |  | 1.33 (1.03–1.72) | 0.027 |
| Subtype |  |  |  |  |  |
| HR+/HER2- | Reference |  |  | Reference |  |
| HR+/HER2+ | 0.58 (0.52–0.65) | <0.001 |  | 0.57 (0.51–0.65) | <0.001 |
| HR-/HER2+ | 0.70 (0.61–0.80) | <0.001 |  | 0.70 (0.61–0.80) | <0.001 |
| HR-/HER2- | 2.18 (1.92–2.47) | <0.001 |  | 2.20 (1.94–2.51) | <0.001 |
| T stage |  |  |  |  |  |
| T1 | Reference |  |  | Reference |  |
| T2 | 1.16 (1.00–1.35) | 0.05 |  | 1.17 (1.00–1.37) | 0.048 |
| T3 | 1.16 (0.98–1.37) | 0.088 |  | 1.17 (0.98–1.39) | 0.076 |
| T4 | 1.34 (1.15–1.56) | <0.001 |  | 1.36 (1.16–1.60) | <0.001 |
| N stage |  |  |  |  |  |
| N0 | Reference |  |  | Reference |  |
| N1 | 0.88 (0.79–0.99) | 0.03 |  | 0.88 (0.78–0.99) | 0.027 |
| N2 | 0.95 (0.81–1.12) | 0.54 |  | 0.94 (0.79–1.11) | 0.467 |
| N3 | 0.88 (0.76–1.02) | 0.087 |  | 0.87 (0.75–1.02) | 0.082 |
| Bone metastases |  |  |  |  |  |
| No/unknown | Reference |  |  | Reference |  |
| Yes | 1.19 (1.08–1.30) | <0.001 |  | 1.21 (1.10–1.33) | <0.001 |
| Brain metastases |  |  |  |  |  |
| No/unknown | Reference |  |  | Reference |  |
| Yes | 1.53 (1.33–1.75) | <0.001 |  | 1.52 (1.32–1.74) | <0.001 |
| Lung metastases |  |  |  |  |  |
| No/unknown | Reference |  |  | Reference |  |
| Yes | 1.30 (1.19–1.43) | <0.001 |  | 1.31 (1.20–1.44) | <0.001 |
| Systemic therapy before surgery |  |  |  |  |  |
| No | Reference |  |  | Reference |  |
| Yes | 0.89 (0.59–1.33) | 0.566 |  | 0.90 (0.60–1.37) | 0.634 |

Abbreviations: PTS, primary tumor surgery; OS, overall survival; BCSS, breast cancer-specific survival; HR, hazard ratio; CI, confidence interval
