# Supplementary material for: Machine learning algorithms for individualized prediction of prognosis in breast cancer liver metastases and the prognostic impact of primary tumor surgery: a multicenter study
Source: Front Endocrinol (Lausanne). 2025 Oct 13;16:1656191. doi: 10.3389/fendo.2025.1656191 (PMC12554575; doi:10.3389/fendo.2025.1656191)
Supplement: Supplementary file 2 [file SupplementaryFile2.docx]

# python 3.8

import os

import sys

import platform

import random

import datetime

import pickle

import math

import json

import hashlib

import pandas as pd

import numpy as np

import matplotlib

# -------------------

GLOBAL_SEED = 123 # 你指定的全局种子

os.environ["PYTHONHASHSEED"] = str(GLOBAL_SEED)

random.seed(GLOBAL_SEED)

np.random.seed(GLOBAL_SEED)

matplotlib.use('AGG')

import matplotlib.pyplot as plt

from sklearn.base import clone, BaseEstimator, TransformerMixin

from sklearn.preprocessing import StandardScaler

from sklearn.inspection import permutation_importance

from sklearn.model_selection import cross_val_predict

from sklearn.model_selection import cross_validate

from sklearn.model_selection import train_test_split as TTS

from sklearn.model_selection import KFold, StratifiedKFold

from sklearn.model_selection import GridSearchCV

from sklearn.pipeline import Pipeline

# 核心5种机器学习算法

from sklearn.linear_model import LogisticRegression

from sklearn.ensemble import RandomForestClassifier, GradientBoostingClassifier

from sklearn.tree import DecisionTreeClassifier

from xgboost import XGBClassifier

# 生存分析相关

from lifelines import KaplanMeierFitter

from lifelines import CoxPHFitter

import AnalysisFunction.X_5_SmartPlot as x5

from AnalysisFunction.X_5_SmartPlot import plot_calibration_curve

from AnalysisFunction.X_5_SmartPlot import calculate_net_benefit

from AnalysisFunction.X_5_SmartPlot import plot_decision_curves

from AnalysisFunction.X_1_DataGovernance import data_standardization

from AnalysisFunction.X_1_DataGovernance import _analysis_dict

from AnalysisFunction.X_2_DataSmartStatistics import comprehensive_smart_analysis

from AnalysisFunction.utils_ml import filtering, dic2str, round_dec, save_fig

from AnalysisFunction.utils_ml import classification_metric_evaluate

from AnalysisFunction.utils_ml import make_class_metrics_dict

from AnalysisFunction.utils_ml import ci

from sklearn.preprocessing import label_binarize

from sklearn.metrics import roc_auc_score, brier_score_loss, roc_curve

import shap

from functools import reduce

plt.rcParams['font.sans-serif'] = ['SimHei']

plt.rcParams['axes.unicode_minus'] = False

from matplotlib import rc

plt.rcParams['ps.useafm'] = True

rc('font', **{'family': 'sans-serif', 'sans-serif': ['FreeSans']})

plt.rcParams['pdf.fonttype'] = 42

# ================== 计数过程构造 ==================

def build_counting_process(

df: pd.DataFrame,

time_col: str,

event_col: str,

id_col: str = None,

exposure_intervals: dict = None,

same_day_event_as_preop: bool = True

) -> pd.DataFrame:

"""

将 (time, event) 生存数据构造成 counting-process 长表 (t_start, t_stop, status, exposure)。

- 如果未提供 exposure_intervals，则每个样本生成单行：t_start=0, t_stop=time, status=event, exposure=0

- 如果提供 exposure_intervals（形如 {id: [(s1,e1,exp1), (s2,e2,exp2), ...]}），

则依据这些区间切分，并在终止区间落点处赋 status。

- 同日手术与事件：若某区间起点与事件时间相同且 same_day_event_as_preop=True，

则事件计入“手术前”区间（即落在上一段区间，或在无上一段时落在 [0, t_event) 区间）。

参数

----

df : 包含至少 time_col, event_col（以及可选 id_col）的 DataFrame

time_col : 生存时间（数值型，单位可自定，需与区间一致）

event_col: 结局指示（1=事件，0=删失）

id_col : 个体ID列名；若 None，则使用 df.index 作为 ID

exposure_intervals : dict，给出每个ID的暴露区间列表：(t_start, t_stop, exposure_flag/int/str)

same_day_event_as_preop : bool，同日事件按术前计入

返回

----

long_df : DataFrame，列含 ['id','t_start','t_stop','status','exposure']

"""

if id_col is None:

ids = df.index

else:

ids = df[id_col]

out_rows = []

for i, row in df.iterrows():

pid = row[id_col] if id_col else i

T = float(row[time_col])

D = int(row[event_col])

# 默认无暴露：单行

if exposure_intervals is None or pid not in exposure_intervals:

out_rows.append(

{"id": pid, "t_start": 0.0, "t_stop": T, "status": int(D), "exposure": 0}

)

continue

# 存在暴露区间

intervals = sorted(exposure_intervals[pid], key=lambda x: (float(x[0]), float(x[1])))

# 先构建不超过 T 的区间

cur_start = 0.0

for (s, e, expv) in intervals:

s = float(s); e = float(e)

if s >= T: # 后续区间都在事件之后，无需加入

break

# 若两个区间之间存在空段，添加空暴露区间

if s > cur_start:

seg_stop = min(s, T)

out_rows.append({"id": pid, "t_start": cur_start, "t_stop": seg_stop,

"status": 0, "exposure": 0})

cur_start = seg_stop

# 添加暴露区间

seg_stop = min(e, T)

out_rows.append({"id": pid, "t_start": cur_start, "t_stop": seg_stop,

"status": 0, "exposure": expv})

cur_start = seg_stop

if cur_start >= T:

break

# 尾段（到 T）

if cur_start < T:

out_rows.append({"id": pid, "t_start": cur_start, "t_stop": T,

"status": 0, "exposure": 0})

# 在事件落点处设置 status

if D == 1:

# 找到包含 T 的段（左闭右开 [t_start, t_stop)；终止时刻归前一段）

# 若存在某段 s==T（同日手术），并且 same_day_event_as_preop=True，则把事件放在上一段

idx_target = None

for j in range(len(out_rows)-1, -1, -1):

if out_rows[j]["id"] != pid:

continue

s = out_rows[j]["t_start"]; e = out_rows[j]["t_stop"]

# 常规：T 落在该段右端点（e==T），事件记在该段

if np.isclose(e, T):

idx_target = j

# 如果段的起点也与 T 相同，说明同日手术起始于 T

if same_day_event_as_preop and np.isclose(s, T):

# 尝试将事件计入上一段

# 找到上一段（同一id且紧邻）

for k in range(j-1, -1, -1):

if out_rows[k]["id"] == pid:

idx_target = k

break

break

if idx_target is not None:

out_rows[idx_target]["status"] = 1

long_df = pd.DataFrame(out_rows)

return long_df

# ================== Cox 特征选择变换器 ==================

class CoxSelector(BaseEstimator, TransformerMixin):

"""

在每个CV训练折中进行：

1) 单因素 Cox 回归（p < p_univ 进入候选）

2) 多因素 Cox 回归（最终保留 p < p_multiv 的变量）

注意：严格使用 fit(X_train_fold) 的索引来子集生存数据，避免信息泄露。

"""

def __init__(self,

survival_times_full: pd.Series,

event_indicators_full: pd.Series,

p_univ: float = 0.10,

p_multiv: float = 0.05,

max_vars: int = None,

penalizer: float = 0.0,

robust: bool = True):

self.survival_times_full = survival_times_full

self.event_indicators_full = event_indicators_full

self.p_univ = p_univ

self.p_multiv = p_multiv

self.max_vars = max_vars

self.penalizer = penalizer

self.robust = robust

self.selected_features_ = None

def _safe_cox_fit(self, df, cols):

"""对 lifelines CoxPHFitter 做鲁棒拟合，必要时回退到带惩罚或去除共线。"""

cph = CoxPHFitter(penalizer=self.penalizer)

try:

cph.fit(df[['__time__', '__event__'] + cols],

duration_col='__time__', event_col='__event__', robust=self.robust)

return cph

except Exception:

cph = CoxPHFitter(penalizer=max(self.penalizer, 0.1))

cph.fit(df[['__time__', '__event__'] + cols],

duration_col='__time__', event_col='__event__', robust=True)

return cph

def fit(self, X: pd.DataFrame, y=None):

if not isinstance(X, pd.DataFrame):

raise ValueError("CoxSelector 需要带索引的 pandas.DataFrame")

# 只取当前训练折的索引对应的生存信息

idx = X.index

t = self.survival_times_full.loc[idx]

e = self.event_indicators_full.loc[idx]

# 组装用于 lifelines 的数据框

df = pd.DataFrame({'__time__': t, '__event__': e}).join(X)

# ---------- 单因素 Cox ----------

univ_keep = []

for col in X.columns:

try:

cph_u = CoxPHFitter(penalizer=self.penalizer)

cph_u.fit(df[['__time__', '__event__', col]], duration_col='__time__', event_col='__event__', robust=self.robust)

pval = cph_u.summary.loc[col, 'p']

if np.isfinite(pval) and pval < self.p_univ:

univ_keep.append(col)

except Exception:

continue

if len(univ_keep) == 0:

univ_keep = list(X.columns)

# 可选：限制候选数量

if self.max_vars is not None and len(univ_keep) > self.max_vars:

pvals = []

for col in univ_keep:

try:

cph_u = CoxPHFitter(penalizer=self.penalizer)

cph_u.fit(df[['__time__', '__event__', col]], duration_col='__time__', event_col='__event__', robust=self.robust)

pvals.append((col, float(cph_u.summary.loc[col, 'p'])))

except Exception:

pvals.append((col, np.inf))

pvals_sorted = sorted(pvals, key=lambda x: x[1])

univ_keep = [c for c, _ in pvals_sorted[:self.max_vars]]

# ---------- 多因素 Cox ----------

final_keep = []

try:

cph_m = self._safe_cox_fit(df, univ_keep)

summ = cph_m.summary

final_keep = [var for var in univ_keep

if var in summ.index and np.isfinite(summ.loc[var, 'p']) and summ.loc[var, 'p'] < self.p_multiv]

except Exception:

final_keep = univ_keep

if len(final_keep) == 0:

pvals = []

for col in univ_keep:

try:

cph_u = CoxPHFitter(penalizer=self.penalizer)

cph_u.fit(df[['__time__', '__event__', col]], duration_col='__time__', event_col='__event__', robust=self.robust)

pvals.append((col, float(cph_u.summary.loc[col, 'p'])))

except Exception:

pvals.append((col, np.inf))

pvals_sorted = sorted(pvals, key=lambda x: x[1])

final_keep = [c for c, _ in pvals_sorted[:max(1, min(3, len(pvals_sorted)))]]

self.selected_features_ = final_keep

return self

def transform(self, X: pd.DataFrame):

if self.selected_features_ is None:

raise RuntimeError("CoxSelector 尚未 fit")

cols = [c for c in self.selected_features_ if c in X.columns]

if len(cols) == 0:

return X

return X[cols]

# ==================（稳健化）==================

def calculate_ipcw_weights(survival_times, event_indicators, prediction_horizon, eps: float = 1e-6):

"""

计算 IPCW 权重（基于删失分布 G(t) 的 Kaplan-Meier 估计）

记 C 为删失时间，则 G(t) = P(C >= t)。权重规则：

- 若个体在 horizon 之前发生事件：w_i = 1 / G(t_i-)

- 若个体在 horizon 之后（或未在 horizon 前发生事件）：w_i = 1 / G(horizon)

- 若个体在 horizon 前被删失：该样本通常不用于 AUC 贡献（在标签构造中已被过滤）

为避免数值不稳定，这里使用下界截断：G(t) = max(G(t), eps)

"""

kmf = KaplanMeierFitter()

censoring_indicators = 1 - np.asarray(event_indicators, dtype=int)

st = np.asarray(survival_times, dtype=float)

try:

kmf.fit(st, event_observed=censoring_indicators)

# 预先计算 G(t) 在关键点的值，减少重复查询

G_h = float(kmf.survival_function_at_times(prediction_horizon).values[0])

G_h = max(G_h, eps)

weights = np.zeros_like(st, dtype=float)

for i, (t, delta) in enumerate(zip(st, event_indicators)):

if t <= prediction_horizon:

if delta == 1:

G_t = float(kmf.survival_function_at_times(t).values[0])

G_t = max(G_t, eps)

weights[i] = 1.0 / G_t

else:

# 在标签构造中，这类个体不会进入有效索引；给0以示占位

weights[i] = 0.0

else:

weights[i] = 1.0 / G_h

except Exception as e:

print(f"IPCW计算错误: {e}")

weights = np.ones(len(survival_times))

return weights

def create_binary_labels_multiple_horizons(survival_times, event_indicators, horizons):

"""

为多个预测时间点创建二分类标签和IPCW权重。

规则：

- 若 t_i <= h 且 delta_i==1 -> label=1（事件发生）

- 若 t_i > h -> label=0（在 h 时刻尚未事件）

- 若 t_i <= h 且 delta_i==0 -> 被删失，剔除（不纳入该 h 的评估）

"""

results_dict = {}

st = np.asarray(survival_times, dtype=float)

ev = np.asarray(event_indicators, dtype=int)

for horizon in horizons:

labels = []

valid_indices = []

for i, (t, delta) in enumerate(zip(st, ev)):

if t <= horizon:

if delta == 1:

labels.append(1)

valid_indices.append(i)

else:

# 删失在h之前：不计入

continue

else:

labels.append(0)

valid_indices.append(i)

weights = calculate_ipcw_weights(st, ev, horizon)

valid_weights = weights[valid_indices]

results_dict[f'{horizon}year'] = {

'labels': np.array(labels, dtype=int),

'weights': valid_weights,

'indices': valid_indices

}

return results_dict

def get_ml_algorithms():

"""

返回研究中使用的5种机器学习算法（统一随机种子）

"""

algorithms = {

'RandomForestClassifier': RandomForestClassifier(random_state=GLOBAL_SEED),

'LogisticRegression': LogisticRegression(random_state=GLOBAL_SEED, max_iter=1000),

'XGBClassifier': XGBClassifier(random_state=GLOBAL_SEED, eval_metric='logloss', n_estimators=100),

'DecisionTreeClassifier': DecisionTreeClassifier(random_state=GLOBAL_SEED),

'GradientBoostingClassifier': GradientBoostingClassifier(random_state=GLOBAL_SEED)

}

return algorithms

def get_hyperparameter_grids():

"""

为5种算法定义详细的网格搜索参数

（保留你原来的网格；注意：部分 sklearn 版本中 'auto' 可能触发警告）

"""

param_grids = {

'RandomForestClassifier': {

'n_estimators': [50, 60, 70, 80, 90, 100, 110, 120, 150, 200, 300, 500],

'max_depth': [3, 5, 6, 7, 8, 9, 10, 15, 20, None],

'min_samples_split': [2, 5, 10, 15, 20],

'min_samples_leaf': [1, 2, 4, 6, 8],

'max_features': ['auto', 'sqrt', 'log2', 0.3, 0.5, 0.7],

'bootstrap': [True, False]

},

'LogisticRegression': {

'C': [0.001, 0.01, 0.1, 0.5, 1.0, 5.0, 10.0, 50.0, 100.0],

'penalty': ['l1', 'l2', 'elasticnet'],

'solver': ['liblinear', 'saga', 'lbfgs'],

'l1_ratio': [0.1, 0.3, 0.5, 0.7, 0.9],

'class_weight': [None, 'balanced']

},

'XGBClassifier': {

'n_estimators': [50, 100, 200, 300, 500],

'max_depth': [3, 4, 5, 6, 8, 10],

'learning_rate': [0.01, 0.05, 0.1, 0.15, 0.2, 0.3],

'subsample': [0.6, 0.7, 0.8, 0.9, 1.0],

'colsample_bytree': [0.6, 0.7, 0.8, 0.9, 1.0],

'gamma': [0, 0.1, 0.2, 0.3, 0.5],

'min_child_weight': [1, 2, 3, 4, 5],

'reg_alpha': [0, 0.01, 0.1, 0.5, 1.0],

'reg_lambda': [0, 0.01, 0.1, 0.5, 1.0]

},

'DecisionTreeClassifier': {

'max_depth': [3, 5, 7, 10, 15, 20, 25, None],

'min_samples_split': [2, 5, 10, 15, 20, 25, 30],

'min_samples_leaf': [1, 2, 4, 6, 8, 10, 12],

'max_features': ['auto', 'sqrt', 'log2', None, 0.3, 0.5, 0.7],

'criterion': ['gini', 'entropy'],

'splitter': ['best', 'random'],

'class_weight': [None, 'balanced']

},

'GradientBoostingClassifier': {

'n_estimators': [50, 100, 200, 300, 500],

'learning_rate': [0.01, 0.05, 0.1, 0.15, 0.2, 0.3],

'max_depth': [3, 4, 5, 6, 7, 8],

'min_samples_split': [2, 5, 10, 15, 20],

'min_samples_leaf': [1, 2, 4, 6, 8],

'max_features': ['auto', 'sqrt', 'log2', None, 0.3, 0.5, 0.7],

'subsample': [0.6, 0.7, 0.8, 0.9, 1.0]

}

}

return param_grids

def calculate_weighted_auc_brier(y_true, y_pred_proba, weights):

"""

计算IPCW加权的AU-ROC和Brier Score

"""

weights = np.asarray(weights, dtype=float)

if weights.sum() > 0:

weights = weights / np.sum(weights) * len(weights)

try:

weighted_auc = roc_auc_score(y_true, y_pred_proba, sample_weight=weights)

weighted_brier = np.average((y_pred_proba - y_true) ** 2, weights=weights)

except Exception as e:

print(f"加权指标计算错误: {e}")

weighted_auc = roc_auc_score(y_true, y_pred_proba)

weighted_brier = brier_score_loss(y_true, y_pred_proba)

return weighted_auc, weighted_brier

def split_datasets(df_seer, df_chinese, features, survival_col, event_col, test_ratio=0.2, random_state=GLOBAL_SEED):

"""

将SEER数据按8:2划分训练/内部测试集，中国医院数据作为外部测试集

"""

seer_features = df_seer[features].dropna()

seer_survival = df_seer[survival_col]

seer_events = df_seer[event_col]

valid_indices = seer_features.index

seer_survival = seer_survival.loc[valid_indices]

seer_events = seer_events.loc[valid_indices]

# Stratified 按事件分层，固定随机数

X_train_seer, X_test_seer, y_survival_train, y_survival_test, y_event_train, y_event_test = TTS(

seer_features,

pd.concat([seer_survival, seer_events], axis=1),

test_size=test_ratio,

random_state=random_state,

stratify=seer_events.loc[valid_indices]

)

y_survival_train, y_event_train = y_survival_train.iloc[:, 0], y_survival_train.iloc[:, 1]

y_survival_test, y_event_test = y_survival_test.iloc[:, 0], y_survival_test.iloc[:, 1]

chinese_features = df_chinese[features].dropna()

chinese_survival = df_chinese[survival_col].loc[chinese_features.index]

chinese_events = df_chinese[event_col].loc[chinese_features.index]

data_splits = {

'train': {

'features': X_train_seer,

'survival_times': y_survival_train,

'event_indicators': y_event_train

},

'internal_test': {

'features': X_test_seer,

'survival_times': y_survival_test,

'event_indicators': y_event_test

},

'external_test': {

'features': chinese_features,

'survival_times': chinese_survival,

'event_indicators': chinese_events

}

}

return data_splits

def _prefix_param_grid(param_grid, prefix):

"""把参数网格键加上前缀，例如 'n_estimators' -> 'clf__n_estimators' """

return {f"{prefix}{k}": v for k, v in param_grid.items()}

def perform_grid_search_with_cv(algorithm, param_grid, X_train, y_train, sample_weights,

cv=10, scoring='roc_auc',

cox_times_full=None, cox_events_full=None,

cox_p_univ=0.10, cox_p_multiv=0.05, cox_max_vars=None,

cox_penalizer=0.0, cox_robust=True):

"""

执行网格搜索+ StratifiedKFold 交叉验证，并在每个训练折中进行 Cox 单/多因素特征筛选

"""

print(f"开始网格搜索 {algorithm.__class__.__name__}...")

print(f"参数搜索空间大小: {np.prod([len(v) for v in param_grid.values()])} 种组合")

# 统一CV为 StratifiedKFold（可复现）

cv_splitter = StratifiedKFold(n_splits=cv, shuffle=True, random_state=GLOBAL_SEED)

# 构造包含 CoxSelector 的 Pipeline

cox_selector = CoxSelector(

survival_times_full=cox_times_full,

event_indicators_full=cox_events_full,

p_univ=cox_p_univ,

p_multiv=cox_p_multiv,

max_vars=cox_max_vars,

penalizer=cox_penalizer,

robust=cox_robust

)

# 注意：参数网格需要前缀到 'clf__'

if algorithm.__class__.__name__ == 'LogisticRegression':

# 处理 Logistic 的 solver-penalty 约束，同时加 'clf__' 前缀

valid_param_grid = []

for penalty in param_grid['penalty']:

for solver in param_grid['solver']:

if (penalty == 'l1' and solver in ['liblinear', 'saga']) or \

(penalty == 'l2' and solver in ['liblinear', 'saga', 'lbfgs']) or \

(penalty == 'elasticnet' and solver == 'saga'):

combo = {

'clf__penalty': [penalty],

'clf__solver': [solver],

'clf__C': param_grid['C'],

'clf__class_weight': param_grid['class_weight']

}

if penalty == 'elasticnet':

combo['clf__l1_ratio'] = param_grid['l1_ratio']

valid_param_grid.append(combo)

best_score = -np.inf

best_model = None

best_params = None

for combo in valid_param_grid:

pipe = Pipeline([

('cox_sel', cox_selector),

('clf', clone(algorithm))

])

try:

grid_search = GridSearchCV(

estimator=pipe,

param_grid=combo,

cv=cv_splitter,

scoring=scoring,

n_jobs=-1,

verbose=0

)

# sample_weight 需要以 'clf__sample_weight' 传入最后一步

grid_search.fit(X_train, y_train, **{'clf__sample_weight': sample_weights})

if grid_search.best_score_ > best_score:

best_score = grid_search.best_score_

best_model = grid_search.best_estimator_

best_params = grid_search.best_params_

except Exception as e:

print(f"参数组合 {combo} 出错: {e}")

continue

cv_scores = best_score

else:

pipe = Pipeline([

('cox_sel', cox_selector),

('clf', algorithm)

])

grid_search = GridSearchCV(

estimator=pipe,

param_grid=_prefix_param_grid(param_grid, 'clf__'),

cv=cv_splitter,

scoring=scoring,

n_jobs=-1,

verbose=1

)

grid_search.fit(X_train, y_train, **{'clf__sample_weight': sample_weights})

best_model = grid_search.best_estimator_

best_params = grid_search.best_params_

cv_scores = grid_search.best_score_

print(f"最优参数: {best_params}")

print(f"交叉验证最优分数: {cv_scores:.4f}")

return best_model, best_params, cv_scores

# ================== manifest 导出 ==================

def _export_manifest_and_grids(save_dir: str, param_grids: dict, cv_splits: int):

os.makedirs(save_dir, exist_ok=True)

manifest = {

"python": sys.version,

"platform": platform.platform(),

"GLOBAL_SEED": GLOBAL_SEED,

"cv": {"type": "StratifiedKFold", "n_splits": cv_splits, "shuffle": True, "random_state": GLOBAL_SEED},

"packages": {

"pandas": pd.__version__,

"numpy": np.__version__,

"matplotlib": matplotlib.__version__,

"scikit_learn": __import__("sklearn").__version__,

"xgboost": __import__("xgboost").__version__,

"lifelines": __import__("lifelines").__version__,

"shap": shap.__version__

}

}

with open(os.path.join(save_dir, "software_manifest.json"), "w", encoding="utf-8") as f:

json.dump(manifest, f, ensure_ascii=False, indent=2)

with open(os.path.join(save_dir, "hyperparameter_grids.json"), "w", encoding="utf-8") as f:

json.dump(param_grids, f, ensure_ascii=False, indent=2)

def _deterministic_run_tag(prefix: str = "run") -> str:

"""

生成确定性 run tag：基于 (GLOBAL_SEED + 当前时间到秒) 的哈希，避免随机数。

"""

ts = datetime.datetime.now().strftime("%Y%m%d%H%M%S")

s = f"{GLOBAL_SEED}-{ts}"

h = hashlib.md5(s.encode("utf-8")).hexdigest()[:8]

return f"{prefix}_{ts}_{h}"

def ML_Classification_Survival(

df_seer,

df_chinese,

features,

survival_col,

event_col,

prediction_horizons=[0.5, 1, 3, 5],

decimal_num=3,

scoring='roc_auc',

n_splits=10,

explain=True,

explain_numvar=5,

explain_sample=2,

searching=True,

savePath=None,

dpi=600,

picFormat='jpeg',

modelSave=True,

randomState=GLOBAL_SEED,

manifest_dir: str = "./supplementary_outputs/",

**kwargs,

):

"""

基于生存数据的多时间点机器学习分类分析

"""

# 导出 manifest 与 超参网格

param_grids = get_hyperparameter_grids()

_export_manifest_and_grids(manifest_dir, param_grids, n_splits)

colors = x5.CB91_Grad_BP

str_time = _deterministic_run_tag("mlsurv")

# 数据划分

data_splits = split_datasets(df_seer, df_chinese, features, survival_col, event_col,

test_ratio=0.2, random_state=randomState)

# 获取5种机器学习算法和参数网格

algorithms = get_ml_algorithms()

# 存储结果

results_dict = {'str_result': {}, 'tables': {}, 'pics': {}, 'save_pics': {}, 'models': {}}

str_result = f"采用5种机器学习方法（Random Forest, Logistic Regression, XGBoost, Decision Tree, GBDT）进行多时间点生存预测分析\n"

str_result += f"预测时间点包括：{', '.join([str(h) for h in prediction_horizons])}年\n"

str_result += f"模型特征包括：{', '.join(features)}\n"

str_result += f"数据集划分：SEER训练集N={data_splits['train']['features'].shape[0]}例，"

str_result += f"SEER内部测试集N={data_splits['internal_test']['features'].shape[0]}例，"

str_result += f"中国医院外部验证集N={data_splits['external_test']['features'].shape[0]}例\n"

str_result += f"参数优化方法：{n_splits}折交叉验证 + 网格搜索（每折训练集内进行 Cox 单、多因素特征筛选，p<0.05 入模）\n\n"

# 对每个时间点和每种算法进行建模

for horizon in prediction_horizons:

str_result += f"=== {horizon}年预测结果 ===\n"

# 为当前时间点创建标签和权重

train_labels_info = create_binary_labels_multiple_horizons(

data_splits['train']['survival_times'],

data_splits['train']['event_indicators'],

[horizon]

)[f'{horizon}year']

internal_labels_info = create_binary_labels_multiple_horizons(

data_splits['internal_test']['survival_times'],

data_splits['internal_test']['event_indicators'],

[horizon]

)[f'{horizon}year']

external_labels_info = create_binary_labels_multiple_horizons(

data_splits['external_test']['survival_times'],

data_splits['external_test']['event_indicators'],

[horizon]

)[f'{horizon}year']

# 获取有效样本

X_train = data_splits['train']['features'].iloc[train_labels_info['indices']]

y_train = train_labels_info['labels']

w_train = train_labels_info['weights']

X_internal_test = data_splits['internal_test']['features'].iloc[internal_labels_info['indices']]

y_internal_test = internal_labels_info['labels']

w_internal_test = internal_labels_info['weights']

X_external_test = data_splits['external_test']['features'].iloc[external_labels_info['indices']]

y_external_test = external_labels_info['labels']

w_external_test = external_labels_info['weights']

# 对每种算法进行建模

horizon_results = {}

best_params_summary = {}

for alg_name, base_alg in algorithms.items():

print(f"训练 {alg_name} for {horizon}年预测...")

# 网格搜索 + StratifiedKFold（每折训练集内做 Cox 特征筛选）

if searching:

best_model, best_params, cv_score = perform_grid_search_with_cv(

algorithm=base_alg,

param_grid=param_grids[alg_name],

X_train=X_train,

y_train=y_train,

sample_weights=w_train,

cv=n_splits,

scoring=scoring,

cox_times_full=data_splits['train']['survival_times'],

cox_events_full=data_splits['train']['event_indicators'],

cox_p_univ=0.10,

cox_p_multiv=0.05,

cox_max_vars=None,

cox_penalizer=0.0,

cox_robust=True

)

clf = best_model

best_params_summary[alg_name] = best_params

else:

cox_selector = CoxSelector(

survival_times_full=data_splits['train']['survival_times'],

event_indicators_full=data_splits['train']['event_indicators'],

p_univ=0.10, p_multiv=0.05, max_vars=None, penalizer=0.0, robust=True

)

pipe = Pipeline([

('cox_sel', cox_selector),

('clf', base_alg)

])

pipe.fit(X_train, y_train, **{'clf__sample_weight': w_train})

clf = pipe

cv_score = None

# 预测

y_pred_internal = clf.predict_proba(X_internal_test)[:, 1]

y_pred_external = clf.predict_proba(X_external_test)[:, 1]

# 计算加权指标

internal_auc, internal_brier = calculate_weighted_auc_brier(y_internal_test, y_pred_internal, w_internal_test)

external_auc, external_brier = calculate_weighted_auc_brier(y_external_test, y_pred_external, w_external_test)

# 存储结果

horizon_results[alg_name] = {

'internal_auc': internal_auc,

'internal_brier': internal_brier,

'external_auc': external_auc,

'external_brier': external_brier,

'model': clf,

'cv_score': cv_score if searching else None

}

cv_info = f", CV分数={cv_score:.3f}" if searching else ""

str_result += f"{alg_name}: 内部测试AUC={internal_auc:.3f}, 外部验证AUC={external_auc:.3f}{cv_info}\n"

# 保存模型

if modelSave:

model_filename = f"{alg_name}_{horizon}year_{str_time}.pkl"

model_path = os.path.join(savePath, model_filename) if savePath else model_filename

with open(model_path, 'wb') as f:

pickle.dump(clf, f)

results_dict['models'][f"{alg_name}_{horizon}year"] = model_filename

# 记录最优参数

if searching:

str_result += f"\n{horizon}年预测最优参数:\n"

for alg_name, params in best_params_summary.items():

str_result += f"{alg_name}: {params}\n"

# 找到最佳模型

best_model_name = max(horizon_results.keys(),

key=lambda x: horizon_results[x]['external_auc'])

best_model = horizon_results[best_model_name]['model']

str_result += f"最佳模型：{best_model_name} (外部验证AUC={horizon_results[best_model_name]['external_auc']:.3f})\n\n"

# 绘制ROC曲线

if savePath:

fig, (ax1, ax2) = plt.subplots(1, 2, figsize=(12, 5), dpi=dpi)

fpr_int, tpr_int, _ = roc_curve(y_internal_test,

best_model.predict_proba(X_internal_test)[:, 1],

sample_weight=w_internal_test)

ax1.plot(fpr_int, tpr_int, 'b-', lw=2,

label=f'Internal Test AUC={horizon_results[best_model_name]["internal_auc"]:.3f}')

ax1.plot([0, 1], [0, 1], 'r--', alpha=0.8)

ax1.set_xlabel('1-Specificity')

ax1.set_ylabel('Sensitivity')

ax1.set_title(f'{horizon}年预测 - 内部测试集ROC ({best_model_name})')

ax1.legend()

ax1.grid(alpha=0.3)

fpr_ext, tpr_ext, _ = roc_curve(y_external_test,

best_model.predict_proba(X_external_test)[:, 1],

sample_weight=w_external_test)

ax2.plot(fpr_ext, tpr_ext, 'g-', lw=2,

label=f'External Test AUC={horizon_results[best_model_name]["external_auc"]:.3f}')

ax2.plot([0, 1], [0, 1], 'r--', alpha=0.8)

ax2.set_xlabel('1-Specificity')

ax2.set_ylabel('Sensitivity')

ax2.set_title(f'{horizon}年预测 - 外部验证集ROC ({best_model_name})')

ax2.legend()

ax2.grid(alpha=0.3)

plt.tight_layout()

roc_filename = save_fig(savePath, f'ROC_{horizon}year', picFormat, fig, str_time=str_time)

results_dict['pics'][f'ROC_{horizon}年'] = roc_filename

plt.close()

# 创建结果表格

results_df = pd.DataFrame({

'算法': list(horizon_results.keys()),

'内部测试AUC': [f"{v['internal_auc']:.3f}" for v in horizon_results.values()],

'内部测试Brier': [f"{v['internal_brier']:.3f}" for v in horizon_results.values()],

'外部验证AUC': [f"{v['external_auc']:.3f}" for v in horizon_results.values()],

'外部验证Brier': [f"{v['external_brier']:.3f}" for v in horizon_results.values()],

'CV分数': [f"{v['cv_score']:.3f}" if v['cv_score'] is not None else "N/A" for v in horizon_results.values()]

})

results_dict['tables'][f'{horizon}年预测结果'] = results_df

# SHAP解释（仅对最佳模型）——占位，按需补充

if explain:

print(f"为{horizon}年最佳模型({best_model_name})生成SHAP解释...")

try:

pass

except Exception:

pass

results_dict['str_result'] = str_result

return results_dict
